# Response of leaf functional traits to soil nutrients in the wet and dry seasons in a subtropical forest on an island

**DOI:** 10.3389/fpls.2023.1236607

**Published:** 2023-12-07

**Authors:** Juanjuan Zhang, Minxia Liang, Sen Tong, Xueting Qiao, Buhang Li, Qiong Yang, Ting Chen, Ping Hu, Shixiao Yu

**Affiliations:** ^1^School of Life Sciences/State Key Laboratory of Biocontrol, Sun Yat-sen University, Guangzhou, China; ^2^Research Institute of Sun Yat-sen University in Shenzhen, Shenzhen, China; ^3^School of Ecology/State Key Laboratory of Biocontrol, Sun Yat-sen University, Shenzhen, China; ^4^Guangdong Neilingding-Futian National Nature Reserve, Shenzhen, China

**Keywords:** fitness, season, leaf functional traits, soil properties, trade-off, subtropical forest

## Abstract

**Introduction:**

Island ecosystems often have a disproportionate number of endemic species and unique and fragile functional characteristics. However, few examples of this type of ecosystem have been reported.

**Methods:**

We conducted a comprehensive field study on Neilingding Island, southern China. The leaf samples of 79 subtropical forest tree species were obtained and their functional traits were studied in the dry and wet seasons to explain the relationships between plant functional traits and soil nutrients.

**Results:**

We found a greater availability of soil moisture content (SMC) and nutrients in the wet season than in the dry season. The values of wet season soil available phosphorus (5.97 mg·kg^−1^), SMC (17.67%), and soil available potassium (SAK, 266.96 mg·kg^−1^) were significantly higher than those of the dry season. The leaf dry matter content, specific leaf weight, leaf density, leaf total carbon, leaf total nitrogen, leaf total calcium, and the N/P and C/P ratios of leaves were all significantly higher in the dry season than in the wet season, being 18.06%, 12.90%, 12.00%, 0.17%, 3.41%, 9.02%, 26.80%, and 24.14% higher, respectively. In contrast, the leaf area (51.01 cm^2^), specific leaf area (152.76 cm^2^·g^−1^), leaf water content (0.59%), leaf total nitrogen (1.31%), leaf total phosphorus (0.14%), and leaf total magnesium (0.33%) were much lower in the dry season than in the wet one. There were significant pairwise correlations between leaf functional traits, but the number and strength of correlations were significantly different in the dry and wet seasons. The SAK, soil total phosphorus (STP), and pH impacted plant leaf functional traits in the dry season, whereas in the wet season, they were affected by SAK, STP, pH, and NO_3_^−^ (nitrate).

**Discussion:**

Both soil nutrients and water availability varied seasonally and could cause variation in a number of leaf traits.

## Introduction

Plant functional traits are physiological, phenological, and morphological features formed by long-term interactions between plant species and the environment. Ecological theory suggests that these evolved traits affect the survival, growth, reproduction, and ultimately the fitness of plants in a given set of environmental conditions (Wright et al., 2005; Díaz et al., 2007; Violle et al., 2007; Díaz et al., 2016; Funk et al., 2017; Laughlin et al., 2020). Leaf traits are associated with the accumulation of biomass and the absorption and utilization of light, water, and nutrients, and they can reflect species’ adaptations to environmental changes and plant growth strategies (Craine et al., 2001; Wright et al., 2004a; Schwoertzig et al., 2016). Additionally, differences in the levels and ratios of these elements can jointly affect plant growth and development, and thus the function and stability of communities and ecosystems (Aerts and Chapin III, 1999).

The growth and development of vegetation depends on interactions between plant functional traits (He et al., 2020). Plants adapt to a changing environment through the co-evolution and trade-off of functional traits (Silvertown, 2004). When two or more plant functional traits are correlated across species and communities, this combination of traits can be considered an “ecological strategy” axis, which allows plants to be arranged along the axis in their most adapted or competitive positions (Wright et al., 2007; Funk et al., 2017). One prime example of this is the leaf economic spectrum (Wright et al., 2004a). Moreover, leaf dry matter content (LDMC) tends to be significantly negatively correlated with N and P in leaves, but seems unrelated to N/P (Wright et al., 2005; Osnas et al., 2013). In general, the leaf total nitrogen (LTN), specific leaf area (SLA), and leaf total phosphorus (LTP) were significantly positively correlated in tropical coastal secondary forests (Yaseen et al., 2022). Accordingly, shifts in plant nutrient utilization strategy should eventually affect nutrient cycling in ecosystems.

Plant functional traits are closely related to environmental factors, and studying their correlations contributes to a better understanding of community-level species coexistence as well as ecosystem functioning. Much research shows that the distribution of plant functional traits is mainly affected by climatic factors on the global or macroscopic scale, land use patterns and disturbances on the mesoscopic scale, and soil and topographic factors on the microscopic or local scale (Reich et al., 1992; Díaz et al., 2001; Wright et al., 2005; Wieczynski et al., 2019). Previous research has indicated that shifts in leaf nitrogen content can be directly caused by changes in the tree basal area, which in turn are directly and positively affected by both island area and soil depth (Schrader et al., 2021). In a recent paper, it was shown that climate correlated significantly with plant leaf functional traits (such as SLA) on the Gran Canaria island (García-Verdugo et al., 2020). Because leaf functional traits are closely related to nutrients in the soil (Craven et al., 2015; Díaz et al., 2016), such traits are usually used as an effective tool for advancing our understanding of the relationship between vegetation and soil (Ordoñez et al., 2009). Many studies have highlighted the fact that soil fertility can alter the trade-off between traits associated with plant growth and nutrient retention (Wright et al., 2004a; Ordoñez et al., 2009; Xu et al., 2020). These two factors play a critical role in shaping species composition and structure of plant communities, with material transformation and interactions between them (Zhang et al., 2017). Plant species can have different effects on soil, thus affecting dynamics of energy and material cycling in soil. Dormann and Woodin (2002) found that the content of nutrient elements in soil could significantly affect leaf functional and reproductive traits in the Arctic region. Moreover, the leaf functional traits of 474 species in 99 sites worldwide are correlated with climate and soil nutrient gradients (Ordoñez et al., 2009). However, the research on functional traits has focused on continental grassland ecosystems and tropical forests, with far less attention being directed to plants of oceanic island ecosystems.

Oceanic islands are geographically isolated by seawater, resulting in limited material/energy cycling and genetic exchange with the mainland. Due to their unique geographical location, limited area, and frequent geological activity, oceanic islands usually host many endemic species, creating island ecosystems that exhibit the characteristics of both terrestrial and oceanic environments (Stuessy et al., 2006). As one of the world’s major habitats of the threatened rhesus macaque (*Macaca mulatta*), Neilingding Island (Guangdong Province, China) harbors an oceanic island ecosystem rich in animal and plant resources. Hence, we conducted an in-depth investigation of relationships between plant functional traits and soil properties on this island. The purpose of this study was to test whether the main soil factors contributing to variations in plant leaf functional traits differed between the wet and dry seasons.

## Materials and methods

### Study site

The field sampling was conducted in subtropical evergreen broadleaved forest within the Neilingding Nature Reserve (22°23′49″–22°25′35″ N, 113°46′18″–113°49′49″ E), an enclosed island located in the east side of Pearl River port in Guangdong Province in southern China, whose total area is ca. 4.98 km^2^ (peak elevation: 340.9 m; **Figure S1**). This nature reserve was established in 1984 to protect the vegetation and several animal species, especially the rhesus macaque. This island has a subtropical monsoon climate with an average annual temperature of 22.0°C and annual precipitation of 1926.9–1975.1 mm, preserving typical native subtropical evergreen broad-leaved forest. There is a pronounced wet season from April to September, in which 85% of the year’s rainfall occurs, and a dry season from October to March that gets much less rain (15%). The island’s dominant vegetation type was changed by local anthropogenic activities but since the nature reserve’s establishment, the native vegetation has been restored rapidly. At present, the dominant tree species are *Mallotus paniculatus*, *Microcos paniculata*, *Phoenix loureiroi*, *Schefflera heptaphylla*, *Ficus microcarpa*, *Pinus massoniana*, *Aporosa dioica*, and *Acacia confusa*. *Mikania micrantha* and *Byttneria grandifolia* are the main invasive plants on Neilingding Island, and their presence has led to serious vegetation degradation. Despite manual removal and chemical control measures, they still wreak havoc on the island every year. Furthermore, the remnants of human activities, such as building debris and agricultural vegetation, are still visible in the landscape.

### Sampling and laboratory analysis

We set up a 15-ha permanent forest plot (300 m × 500 m; **Figure S1**) in the reserve on Neilingding Island and divided the plot into 375 quadrats (each 20 m × 20 m). We then surveyed and tagged all live tree individuals with diameter at breast (DBH) ≥ 1 cm, determined their species identity, and measured their heights from December 2019 to January 2020.

In this study, 79 tree species, representing > 75% of all plant species in the 15-ha permanent plot, were chosen for sampling to adequately represent the forest community (**Table S8**). Fully sun-exposed and mature leaves of 1–20 adult individuals of each tree species were randomly collected from the canopy top across the 15-ha plot in both the dry (January) and wet (August) seasons of 2020. Leaves with obvious evidence of substantial mechanical damage or biotic alteration (e.g., leaves with insect damage or disease) were not sampled. For each individual, five of its sampled leaves per species were scanned to obtain leaf area (LA) and leaf thickness (LT), and they were weighed to measure their fresh mass (FM) and dry mass (DM) before and after oven-drying at 65°C. For each species’ leaf sample, SLA was calculated as the ratio of leaf area to leaf dry mass and LDMC as the ratio of leaf dry mass to fresh mass (Garnier et al., 2001). Leaf volume (LV) was calculated as the LT x LA. The leaf water content (LWC) was calculated as the ratio of leaf fresh mass minus leaf dry mass to leaf fresh mass, while leaf density (LD) was calculated as the ratio of leaf dry mass to leaf volume. All leaf samples of each species were oven-dried at 65°C to a constant weight before being ground and mixed thoroughly to analyze their elements. Total leaf C and N concentrations (% of dry mass) were analyzed using an elemental analyzer (Vario MAX cube elemental analyzer; Elementar, GER). Total leaf P, K, Ca, and Mg concentrations (% of dry mass) were analyzed using the molybdate/ascorbic acid method after H_2_SO_4_-H_2_O_2_ digestion (Poorter and Navas, 2003).

Meanwhile, soil samples were collected at a depth of 0–20 cm. To do this, five soil cores were collected from each of the three quadrats with a soil auger and then bulked on a per-quadrat basis to form one composite sample. Soil water content was determined in the laboratory. The complete series of soil properties were evaluated on the dry weight basis. The soil samples were air dried, mill ground, and selected with a 0.25 mm sieve, all performed at 25°C, before subjected to physicochemical analysis. The soil moisture content (SMC) was determined using a 20-g subsample of the fresh soil (oven dried at 105°C for 24 h). With the potentiostatic method, soil pH was measured in a 1:5 (w/v) soil to water suspension. Soil available phosphorus (SAP) was extracted with 0.5 mol L^−1^ NaHCO_3_ and determined through the molybdate colorimetric test (the Olsen method) with a UV-VIS spectrophotometer (UVmini-1240, SHIMADZU). The extraction of soil NO_3_^−^-N and NH_4_^+^-N was performed with 2 mol L^−1^ KCl, and their levels were determined with a flow injection auto analyzer (FIAstar 5000 Analyzer, Foss Tecator, Denmark). Soil alkali-hydrolyzable nitrogen content was quantified by the method of Roberts et al. (2011). Briefly, 5 g samples of soil were distilled with 2 mol L^−1^ NaOH for 5 h and then with 10 mol L^−1^ NaOH for 7 min. Boric acid (40 g L^−1^) was used to absorb the liberated NH_3_ via direct steam distillation. The soil alkali-hydrolyzable nitrogen content was quantified by a conductometric titration. Soil organic carbon (SOC) was measured using the Walkley-Black method (Bottomley et al., 2020). Available P concentrations in the extracts (2.5 g air-dried soil was extracted with 50 ml of 0.5 M NaHCO_3_) were measured using the molybdate blue colorimetric method. We determined soil total nitrogen (STN) content with an elemental analyzer (Vario MAX cube elemental analyzer, Elementar, GER). The soil total phosphorus (STP) contents were analyzed using the molybdate/ascorbic acid method after H_2_SO_4_–HClO_4_ digestion and determined with a spectrophotometer (John, 1970; Hou et al., 2013; Zhang Y. et al., 2022). In the last step, soil available potassium (SAK) was quantified by applying the neutral normal ammonium acetate extraction method (Arif et al., 2018). To 5 g air-dried soil, 50 ml of 1 M ammonium acetate solution (pH 7) was added, and the mixture was shaken for a half hour (275 rpm) and then allowed to settle. The extract from the soil solution suspension was filtered (Arif et al., 2018). The SAK concentration of extract was measured by flame spectrometry (Arif et al., 2018) (Flame photometer PFP7, JenWay, England).

### Statistical analysis

All species in a given 20 m × 20 m quadrat were used to calculate the plot-level community-weighted mean (CWM), which is the species abundance-weighted mean of a trait at the plot level as calculated from the trait values at the species level (Garnier et al., 2004; Eichenberg, 2014). Plot-level leaf functional traits are reported. The paired *t* test was used to compare leaf functional traits and soil physicochemical properties between the dry and wet seasons. Pearson correlation analysis was performed for the functional traits of plant leaves in both the dry and wet seasons, separately.

The relationship comparison between the dry and wet seasons for each possible pairwise combination of leaf functional traits was proceeded by standardized major axis (SMA, data were log10-transformed for analysis) regression (Warton et al., 2006; Leishman et al., 2007). In the case of describing the bivariate scatter of two traits, SMA regression estimates lines with greater precision than major axis regression (Warton et al., 2006; Leishman et al., 2007). SMA regression also finds the best-fit scaling relationship between pairs of traits on log–log axes (Leishman et al., 2007). In comparing the cloud of points that describe the pairwise relationship of traits from the dry and wet seasons, the slope of the line of best fit may vary; in some situations, the slopes may completely overlap, shift along the common slope relative to each other, and/or shift in one dimension only, resulting in elevation differences (Leishman et al., 2007). SMA slopes were fitted for each season and tested for homogeneity. If a common slope was obtained because homogeneity was true, the elevation differences were then tested (Leishman et al., 2007). The software SMATR (Standardized Major Axis Tests and Routines) was used for the SMA regression analyses (significance level: α = 0.05) (Falster et al., 2006; Leishman et al., 2007).

Principal component analysis (PCA) was used to identify the important traits in the dry and wet seasons. Finally, redundancy analysis (RDA) was conducted to determine the relationships between leaf functional traits and soil physicochemical properties in the dry and wet seasons. All statistical analyses were implemented in the R v4.0.5 (R Core Team) software computing platform.

## Results

### Comparison of soil properties and leaf functional traits for the dry and wet season

**Figure 1:**
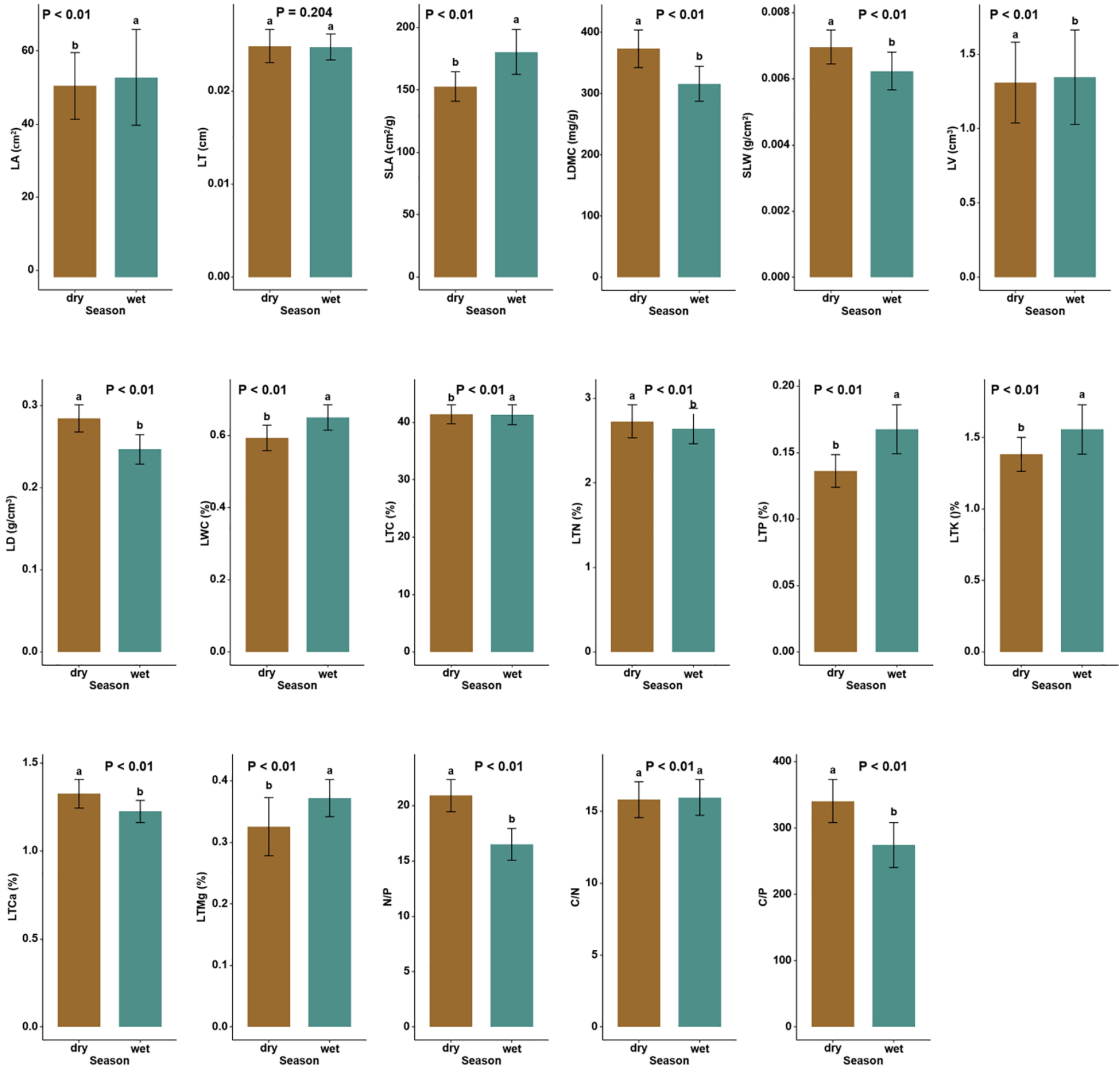
Differences in the CWM (community-weighted mean) leaf functional traits between the dry and wet seasons in Neilingding Island (P < 0.05); lettering is used to indicate significant differences (P < 0.05) between leaf functional traits in dry versus wet seasons.

The soil physicochemical properties, such as SAP, SMC, SAK, SOC, and STP, were significantly larger in the wet than in the dry season by 173.85%, 123.95%, 56.49%, 15.83%, and 6.82%, respectively (**Table 1**, *P* < 0.05). Among leaf functional traits, the leaf N/P was higher in the dry season (26.78% increase); as was C/P, LMDC, specific leaf weight (SLW), LD, leaf total carbon (LTC), LTN, and leaf total calcium (LTCa), which increased by 24.14%, 18.06%, 12.90%, 12.00%, 0.17%, 3.41%, and 9.02%, respectively (**Tables S1, S2**; **Figure 1**; *P* < 0.05). In contrast, LTP, leaf total potassium (LTK), leaf total magnesium (LTMg), LA, SLA, LV, LWC and leaf C/N were significantly lower in the dry season, being respectively reduced by 17.64%, 11.54%, 10.81%, 4.30%, 15.34%, 2.97%, 9.23%, and 0.94% (**Tables S1, S2**; **Figure 1**; *P* < 0.05).

**Table 1:** Differences in soil properties between the dry and wet seasons in Neilingding Island (P < 0.05).

Season	SMC (%)	pH	SOC (g kg^−1^)	STN (g kg^−1^)	STP (g kg^−1^)	SAP (mg kg^−1^)	SAK (mg kg^−1^)
Dry season	7.89 ± 2.4^b^	4.6 ± 0.42^a^	28.75 ± 7.3^b^	1.32 ± 0.23^a^	0.44 ± 0.08^b^	2.18 ± 2.27^b^	170.59 ± 47.02^b^
Wet season	17.67 ± 1.82^a^	4.64 ± 0.32^a^	33.3 ± 10.24^a^	1.32 ± 0.37^a^	0.47 ± 0.12^a^	5.97 ± 3.45^a^	266.96 ± 87.77^a^

Different letters within each row indicated significant differences (P < 0.05) in the paired t tests of soil properties in the dry versus wet season. Values are the mean ± SD. SMC, soil moisture content; SOC, soil organic carbon; STN, soil total nitrogen; STP, soil total phosphorus; SAP, soil available phosphorus; SAK, soil available potassium.

### Correlations among leaf functional traits

**Figure 2:**
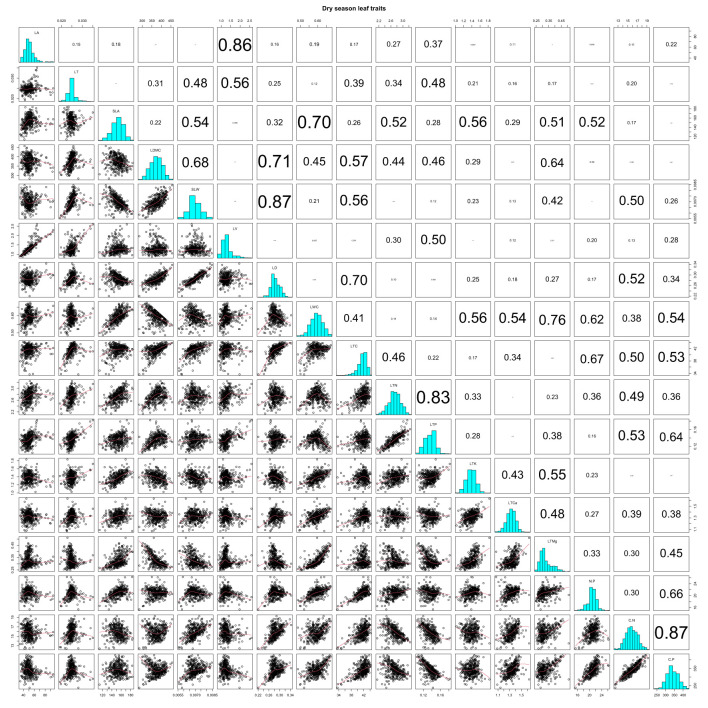
Pearson correlations of CWM leaf functional traits in the dry season on Neilingding Island.

LA and LT showed highly significant positive correlations with LV in the dry season (*r* = 0.86 and 0.56). SLA was negatively correlated with SLW (*r* = −0.54), but it SLA was positively correlated with LWC, LTN, LTK, LTCa, LTMg, and N/P (*r* = 0.70, 0.52, 0.56, 0.51, and 0.52; **Table S3**; **Figure 2**; all *P*-values < 0.001). Correlations of LDMC with SLW, LD, and LTC were strongly positive (*r* = 0.68, 0.71, and 0.57), but they were negative for LTMg (*r* = −0.64) in the dry season (**Table S3**; **Figure 2**; all *P*-values < 0.001). We found highly significant positive correlations of SLW with LD, LTC, and C/N (*r* = 0.87, 0.56, and 0.50), and likewise between LTN and LTP (*r* = 0.83). However, LTP was negatively correlated with both C/N and C/P (*r* = −0.53 and −0.64) (**Table S3**; **Figure 2**; all *P*-values < 0.001).

**Figure 3:**
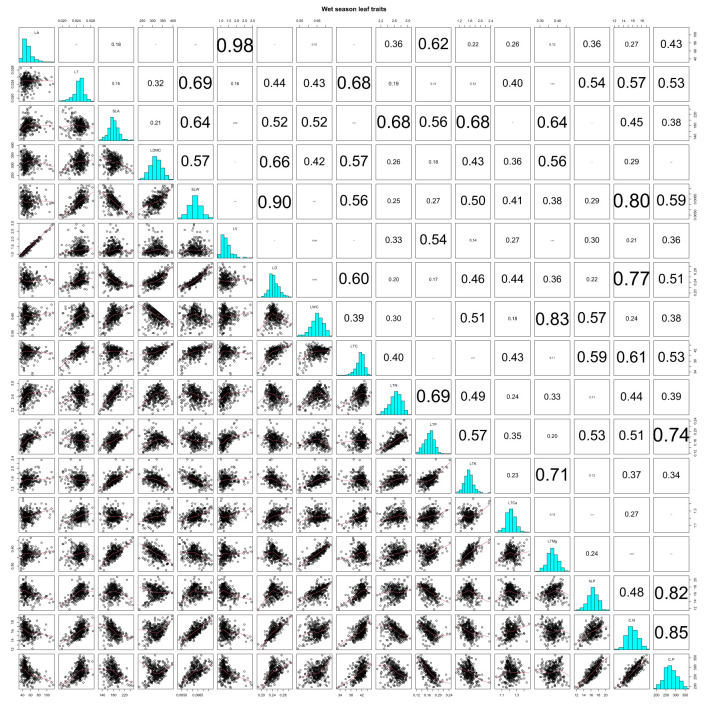
Pearson correlations of CWM leaf functional traits in the wet season on Neilingding Island.

In the wet season, LA had rather strong positive correlations with LV and LTP (*r* = 0.98 and 0.62), as did LT with SLW, LTC, N/P, C/N, and C/P (*r* = 0.69, 0.68, 0.54, 0.57, and 0.53) (**Table S3**; **Figure 3**; all *P*-values < 0.001). SLA decreased with SLW and LD (*r* = −0.64 and −0.52), but increased with LWC, LTN, LTP, LTK, and LTMg (*r* = 0.52, 0.68, 0.56, 0.68, and 0.64) (**Table S3**; **Figure 3**, all *P*-values < 0.001). LDMC showed positive correlations with SLW, LD, and LTC, (*r* = 0.57, 0.66, and 0.57) and a negative correlation with LTMg in the wet season (*r* = −0.56) (**Table S3**; **Figure 3**; all *P*-values < 0.001). SLW increased with LD, LTC, C/N, and C/P, with the correlation being strongest for LD and C/N (*r* = 0.90 and 0.80), but SLW decreased with LTK (*r* = −0.50), while LTN and LTP increased in tandem (*r* = 0.69) (**Table S3**; **Figure 3**; all *P*-values < 0.001). LTP’s correlation was highly positive with LTK, though it was negative with N/P, C/N, and C/P (*r* = 0.57, −0.53, −0.51, and −0.74) (**Table S3**; **Figure 2**; all *P*-values < 0.001).

In both seasons, there were highly significant positive correlations between LD and LTC (*r* = 0.70 and 0.60), and likewise between LWC and LTMg (*r* = 0.76 and 0.83) (**Tables S2, S3;** **Figures 2**, **3**; all *P*-values < 0.001). LTC showed strong positive correlations with N/P, C/N, and C/P in both seasons (**Tables S3, S4**, **Figures 2**, **3**; all *P*-values < 0.001). There were highly significant positive correlations between N/P, C/N, and C/P in the dry and wet seasons. The correlation coefficients for N/P and C/P were 0.66 and 0.82 in the two seasons, which was lower than the 0.87 and 0.85 for C/N and C/P (**Tables S3, S4**; **Figures 2**, **3**; all *P*-values < 0.001). In both seasons, LTK and LTMg were positively correlated (*r* = 0.55 and 0.71), as were LTCa and C/N; however, LTCa had weakly positive correlations with N/P and C/N in the dry season (**Tables S3, S4**; **Figures 2**, **3**; all *P*-values < 0.001).

In addition, pairwise comparisons were examined for all leaf functional traits. The results presented significant correlations for most pairwise leaf functional trait relationships in both seasons (**Table S5**; **Figure S2**). Additionally, significant differences in the linear regression slope (59 pairwise combinations) between dry and wet seasons were observed for leaf functional traits, and clear shifts along a common slope (32 pairwise combinations) were present (**Table S5**; **Figure S2**).

### PCA of leaf functional traits

**Figure 4:**
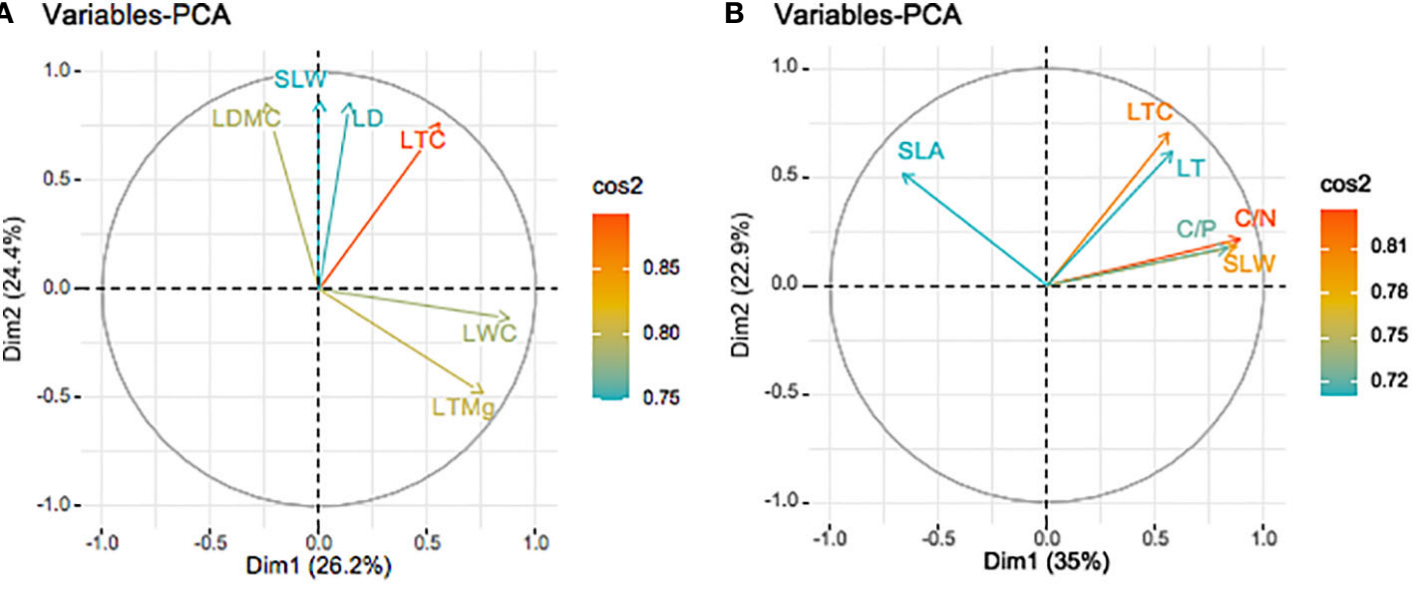
Biplot of principal components and CWM leaf functional traits in the dry **(A)** and wet **(B)** seasons on Neilingding Island.

The PCA of 17 leaf functional traits showed high communalities in the dry season. The leaf functional trait with the highest and lowest communality was LTN (0.98) and LTCa (0.656) respectively (**Table S6**). Five principal components with eigenvalues >1 (4.462, 4.146, 3.632, 1.763, and 1.194) were extracted, which together explained 89.39% (>85%) of the trait variance (26.25%, 24.39%, 21.37%, 10.37%, and 7.02%, respectively; **Table S6**). The variables in the PCA are expressed via the square cosine (cos^2^), with a high value indicating that a variable makes a great contribution and is located near the correlation circle. Initial factors with correlation coefficients >0.75 were selected from the rotated component matrix (**Figure 4A**). The first two axes explained 50.6% of the variance (**Figure 4A**). Six key initial factors were extracted and their contribution to the first and second principal components, and were ranked as LTC > LTMg > LDMC > LWC > SLW > LD (**Figure 4A**).

PCA was performed on 17 leaf functional traits in the wet season, and high communalities were also obtained. The trait with the highest communality was LDMC (0.63), while that with the lowest communality was LTCa (0.523), which indicates little loss of information during factor categorization. Four principal components with eigenvalues >1 (5.947, 3.893, 3.237, and 1.498) were extracted; they explained 85.73% (>85%) of the variance (34.98%, 22.90%, 19.03%, and 8.81%, respectively; **Table S7**). Initial factors with correlation coefficients >0.7 were selected from the rotated component matrix. The first two axes explained 52.9% of the variance. Six key initial factors were extracted and their contribution to the first and second principal components and were ranked as follows: C/N > LTC > SLW > C/P > SLA > LT (**Figure 4B**).

### Effects of environmental factors on functional traits

**Figure 5:**
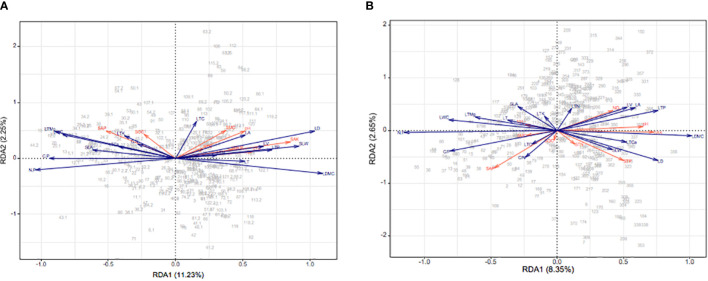
The RDA biplot of environmental factors and CWM leaf functional traits in the dry **(A)** and wet **(B)** seasons on Neilingding Island; red lines are environmental factors and blue lines are leaf functional traits.

The first and second RDA axes jointly explained 13.48% of the variance (**Figure 5A**). The first axis in particular captured the relationships between leaf functional traits and soil properties, and mainly represents the effects of SAP, SMC, pH, STN, SAK, available N, and STP, while the second axis mainly describes the effect of SOC. Notably, STP was positively correlated with LTP and SLW; likewise, for SAK with LV, soil pH and SMC with LA, and SAP with LTK (**Figure 5A**). In the wet season, the first and second axes jointly explained 11.0% of the variance (**Figure 5B**). Both axes, especially the first one, demonstrated the relationships between leaf functional traits and soil properties. The first axis mainly described the effects of SAP, SMC, NH_4_^+^, NO_3_^−^ pH, SAK, and STP, while the second axis showed the effects of SOC and STN. Both SAK and pH were positively correlated with LDMC, was NO_3_^−^ with LV, and STP with SLW.

## Discussion

### Soil water and nutrient availability

SOC, STN, STP, SAP, and SAK are important indicators of soil fertility and they can directly impact soil quality. However, soil pH was similar between dry and wet seasons, and the soil in the plot was slightly acidic (**Table 1**). Due to the drastic wet and dry seasonal differences on Neilingding Island and the notable seasonal precipitation variation, SMC was greater in the wet than dry season (**Table 1**). Temperature and humidity are important environmental and climatic factors that affect the organic carbon in soil (Davidson et al., 2000; Zhang Y. et al., 2022; Cheng et al., 2023). Accordingly, the SOC was higher in the wet than in the dry season (**Table 1**), which was consistent with Xiao et al. (2020). The previous studies reported that the total plant biomass and the fraction of biomass allocated to roots increased with high temperature in subtropical forests (Leon and Joseph, 2009; Li et al., 2017). Therefore, root growth rates might increase with soil temperature (Kaspar and Bland, 1992; Malhotra et al., 2020). Moreover, Post et al. (1982) estimated the global carbon storage, concluding that SOC increased with more precipitation, which is consistent with our results. The greater temperature and precipitation in the wet season of Neilingding Island improves the plants’ photosynthesis and their utilization of available nutrients in soil, which in turn enhances their growth and organic matter secretion, and thus the SOC. We found that the STP was higher in the wet than in the dry season (**Table 1**), whereas STN was similar across seasons. This pattern differed from meadow grasslands studied by Yang et al. (2021), in which STN and STP increased significantly and slightly with precipitation in the dry and wet years, respectively. Research on boreal forests has shown that drought reduces the STN, but negligibly affects total soil P (Zhang et al., 2020). Such a discrepancy among studies could be explained by the various types of ecosystems they investigated. Moreover, our results for a subtropical forest show that SAP is significantly lower in the dry than wet season (**Table 1**). However, for a Mediterranean shrubland, there was no apparent effect of drought on soil available P (Sardans et al., 2006). However, the previous study reported that increased precipitation significantly increased soil microbial biomass P, which lead to increased soil organic P in a temperate forest (Zhang et al., 2020). Water is a key factor affecting soil K uptake and release, but in that Mediterranean shrubland non-significant effects of drought on total soil K, extractable K, and non-extractable K were found. This is inconsistent with our results that SAK is significantly lower in the dry season than wet season (**Table 1**).

### Trade-offs between plant functional traits

The levels and ratios of leaf C, N, and P indicate the strategy of nutrient utilization in plant growth. In the present study, LTN, LTP, and N/P in the dry and wet seasons were higher than those (1.41%, 0.088%, and 15.2%) of 753 terrestrial plants in China (Han et al., 2005). Our analysis showed that LTC was higher in the dry than wet season, which was inconsistent with the previous study reported by Sardans and Peñuelas (2008). A global-scale study found that leaf N increased with decreasing precipitation (Wright et al., 2004a), which is in line with our result of LTN being significantly higher in the dry season than wet season. Meanwhile, a somewhat higher LTC and much higher LTN in the dry season directly led to an insignificant seasonal difference in leaf C/N (**Table S1**; *P* < 0.05). According to the growth rate hypothesis, plants with high P, low C/P, and low N/P usually have high growth rates, and vice versa (Elser et al., 1996; Elser et al., 2003). Bertiller et al. (2005) found that drought reduced the P in shrub leaves. We found that LTP was significantly lower in the dry than wet season, while the opposite characterized N/P and C/P (**Table S1**; **Figure 1**). Hence, forest plants on Neilingding Island exhibit higher growth rate and light capture efficiency in the wet season than in the dry season (Liu et al., 2014). Meanwhile, N/P was > 20 in the dry season, indicating that plant growth was severely restricted at that time by P, but it fell to 16.49 in the wet season. This significant reduction suggests that SAP increases during the wet season to alleviate P deficiency (Koerselman and Meuleman, 1996; Güsewell, 2004). Mineral nutrients in plant leaves can vary significantly with the amount of water in soil. The LTK was lower in the dry than wet season, which may be explained by latter’s greater soil available K.

The wet season also featured a higher SLA and LWC, but a lower LMDC, SLW, and LD (**Table S2**; **Figure 1**). Plant communities with conservation strategies usually have a high LDMC (Domínguez et al., 2012; Wang et al., 2023). Accordingly, we suppose that a conservation strategy was adopted during the dry season, whereas the wet season facilitated plant leaf construction and thus photosynthetic efficiency. By analyzing the LA of 7670 species worldwide, Wright et al. (2017) showed that plant leaves were smaller under drought conditions but larger in areas with elevated temperature, water availability, and sunlight, which is consistent with our findings (**Table S1**; **Figure 1**). The low SLW in the dry season led to greater resource acquisition per unit leaf area, thereby promoting plants’ growth under stressful conditions and their adaptation to the local environment.

LT is an informative plant trait because it is closely related to the uptake of resources and water assimilation and conservation (Reich et al., 1998; Meziane and Shipley, 1999; Liu et al., 2008; Afzal et al., 2017). In the present study, compared with the wet season, leaves showed higher SLW, lower SLA, and higher LDMC in the dry season, indicating greater resource uptake and conservation at this time in the face of limited nutrients. This enhanced resource utilization and could help cope with the scarcity of soil water and nutrients caused by the low temperatures and reduced precipitation during a dry season (Poorter and Garnier, 2007). LT measures the distance that water diffuses from the leaf interior to the surface. Plants with a low SLA usually have a high LT, but a low LA (Abid et al., 2016; Wellstein et al., 2017; Wright et al., 2017; Zhang C. et al., 2022). This is inconsistent with our result of SLA being much lower in the dry than the wet season and LT being similar between seasons (**Table S1**; **Figure 2**). It is generally accepted that plants with low SLA and high LV are efficient in their conservation and utilization of resources (Reich et al., 1998; Wilson et al., 1999); yet, for the subtropical forest island we studied, their trees’ LV was similar between dry and wet seasons.

### Correlations between leaf functional traits

Leaf functional traits are closely correlated, and plants respond to environmental changes via combinations of these traits. Plant trait correlations represent trait coordination/trade-off and environmental adaption strategies during plant growth, development, and reproduction (Stearns, 1989; He et al., 2009; Freschet et al., 2015). Since leaf functional traits are sensitive to environmental and climatic changes, it is not surprising to find differences in the correlations between these traits under differing growth conditions. Here, the correlations between LA and LV were the strongest in both dry and wet seasons, especially in the latter (there was a greater rise in LV with increasing LA during the wet season, which bolstered photosynthate accumulation and storage). That LA in the wet season was high and also positively correlated with both SLA and LTP, yet negatively correlated with C/N and C/P, implies greater plant growth rates in the wet season (Wright et al., 2004a).

More than 20 years ago, a strong correlation between SLA and LDMC was reported (Poorter and De Jong, 1999), which our data supported as well (**Figures 2**, **3**; **Tables S3, S4**). Furthermore, the positive correlation between SLA and LTN in the wet season suggested that a high SLA (low SLW), large light absorption area, and high LTN enabled a high net photosynthetic rate and high growth rate of plants (Wright and Westoby, 2000; Wright et al., 2001; Wright et al., 2004b; Kang et al., 2021). In the dry season, the low SLA (high SLW) suggested that a large proportion of leaf material was used for building defense structures or increasing the density of mesophyll cells, forming thick and small leaves to increase the distance and resistance of water diffusion from the leaf interior to the surface and to lessen water losses from plants (Reich et al., 1998; Wright et al., 2004a).

Plant ecological behavior and resource acquisition can be measured by LDMC, in that a low LDMC indicates high resource acquisition (Poorter et al., 2008; Wang et al., 2023). In the subtropical forest studied here, 79 evergreen broad-leaved tree species in both seasons had positive correlations between LDMC and SLW, a result consistent with previous studies (Lambers and Poorter, 1992; Grime et al., 1997). SLW is a fundamental indicator of plant leaf structure and how species have adapted to the environment during their evolution (Reich et al., 2003). Herein, we found a highly significant positive correlation between SLW and LD indicates that a high LD is associated with successful adaptation to resource-poor habitats. Wright et al. (2005) concluded that LTK is weakly correlated with key plant growth indicators such as photosynthetic rate, SLW, and leaf longevity, and that LTK is not a key factor affecting plant growth. However, we uncovered a highly significant negative correlation between LTK and SLW, and SLW decreased with increasing K per unit leaf area. LD serves as an indicator of self-protection and nutrient allocation/retention in plants. In our study, there were highly significant positive correlations between LD and LTC in the dry and wet seasons, implying that C sequestration increased with the increasing density of leaf tissues. Moreover, the highly significant positive correlations between LWC and LTC in both seasons suggest LWC is closely tied to photosynthesis, and that photosynthetic efficiency improves with greater LWC.

Carbon is an essential element in plant structure and biological macromolecules. Because C acts in concert with N and P during processes of growth, development, and reproduction in plants, they are usually positively associated. In the present study, LTC featured highly significant positive correlations with LTN, LTCa, N/P, C/N, and C/P in either season, with a highly significant positive correlation between leaf LTC and LTP in the dry season. In plants, N, P, and K have synergistic effects and are usually positively correlated with each other (Wright et al., 2005), a view supported by our findings from the subtropical forest. We also found highly significant positive correlations of LTN with LTP, LTK, and N/P in the dry season, and between LTN, LTP, and LTK in both seasons, with the strongest correlation between LTN and LTP, which is consistent with several studies (Reich et al., 2003; Han et al., 2005; Wright et al., 2005; Zhang et al., 2018). The main reason for the highly significant positive correlation between LTN and LTP is that N and P, as widely limiting elements for plant growth and development, are coupled with many physiological and biochemical processes (Niklas, 2006; Zhang et al., 2018; Wen et al., 2022). Therefore, they remained relatively stable despite a seasonally changing environment (Fonseca et al., 2000; Güsewell, 2004). Furthermore, N/P, C/N, and C/P display highly significant positive correlations in the dry and wet seasons. The correlation coefficients of N/P and C/P in the dry and wet seasons were 0.66 and 0.82, respectively, which were lower than those between C/N and C/P (0.87 and 0.85). Our results support the commonly observed strong correlations between plant functional traits (He et al., 2009; Li et al., 2022). The functional traits of leaves for trees in the plot were tightly correlated in both seasons, but the number and strength of the correlations were greater in the wet season. Meanwhile, LTN and LTP were correlated with SLA, LA, LT, and LV, implicating a pivotal role for nutrients in shaping the species’ leaf morphology of the plant community on the island.

LTC and SLW with high loadings in the dry and wet seasons were identified as the primary leaf functional traits at the study site. Leaf organs are responsible for C sequestration in plants, and C is the most abundant element in these tissues (Mengel et al., 2001). LTC usually reaches ~40% and is involved in the formation of structural materials in plants, and it has an important role in maintaining their growth, development, and metabolism. SLW is a measurement of leaf photosynthetic performance because it is closely related to photosynthesis; accordingly, SLW can be used to gauge the productivity of individual plants as well as plant communities. Low SLW means there is a high resource acquisition per unit leaf area, which facilitates plants’ growth under adverse conditions and improves their adaptation to the environment. Therefore, LTC and SLW are important leaf functional traits that affect the composition and structure of plant communities in the dry and wet seasons, being crucial leaf functional traits in either season on the island.

### Correlations of leaf functional traits and environmental factors

Soil is the chief source of water and nutrients essential for plant growth and development, which can be directly impacted by the variation in and distribution of water and nutrients in soil. Our results demonstrate that soil nutrients, water, and pH could impact the functional traits of leaves in the dry season. At this time, available N, P, K, and water in soil and soil pH may be the factors limiting plant growth through their impact on leaf functional traits. In the wet season, however, it is only soil nutrients and pH that influence leaf functional traits, as water no longer limits plant growth. A comprehensive analysis using global climate and soil properties established that soil nutrients are the factors affecting leaf functional traits, with precipitation being the major climatic factor affecting the balance between growth-related leaf traits and soil fertility (Ordoñez et al., 2009). The RDA revealed that SAP, SMC, pH, STP, and SAK affected leaf functional traits in the dry season. Among them, STP and SAP showed stronger correlations with leaf functional traits in the dry than wet season, perhaps due to P-deficient soil in the dry season. Research has shown that plant functional traits are sensitive to changes in P due to the lack of this element in most tropical forest soils (Baribault et al., 2012). Previous studies have found that soil P is the main limiting factor for plant growth and development in Southeast Asian tropical forests (Vitousek and Sanford Jr, 1986; Allen et al., 2020). Our study’s results suggested that P may restrict plant growth by affecting leaf functional traits in both dry and wet seasons; STP and SAP were significantly lower in the dry season than in the wet season; and P restriction was more significant in the dry season (N/P > 20; **Table S2**). Regarding SMC, it is distinguished as a major limiting factor for plant growth and development in arid regions (Wang et al., 2015). For the subtropical forest, we found that SMC is significantly lower in the dry season (only 44.65% of that in the wet season, **Table 1**). Moreover, SMC increases with LA in the dry season, indicating that plants may adapt to water stress by regulating LA. In the dry season, water in soil could modulate plant growth by reducing LA and thus photosynthesis. Other work has reported that soil pH could affect the species composition and functional traits of plant communities (Tahmasebi Kohyani et al., 2008; Maes et al., 2020). We found a positive correlation between soil pH and LDMC in the wet season, indicating that the accumulation of nutrients in plants was regulated by soil pH. Altogether, leaf functional traits exhibit different correlations with soil properties in the dry versus wet seasons, and these differences are closely linked to soil nutrients and water content.

## Conclusions

According to this field study, soil water and nutrient availability are significantly higher in the wet than in the dry season in the 15-ha permanent forest plot on Neilingding Island. We compared plant leaf functional trait associations in the two seasons and found that significant relationships existed in the dry season, with stronger trait–trait associations in the wet season. Acquisitive traits indicate a higher competitive ability and faster resource acquisition for tree species in the wet season. Plant growth rates are lower in the dry than wet season due to severe P deficiency. The functional traits of plant leaves on Neilingding Island are closely correlated with each other, but the number and strength of these correlations varied seasonally, being generally stronger in the wet season. Distinct leaf functional traits were prominent in the dry and wet seasons. Both LTC and SLW, with high loadings in the dry and wet seasons, could be used as the key leaf functional traits in either season. Furthermore, leaf functional traits responded differentially to soil properties during the dry and wet seasons. This study provides valuable insights into the mechanisms by which environmental factors influence plant functional traits in coastal island ecosystems. Our empirical findings lay a theoretical foundation for maintaining the stability of plant community composition and ecosystem functioning on coastal islands.

## Data availability statement

The raw data supporting the conclusions of this article will be made available by the authors, without undue reservation.

## Author contributions

SY and JZ conceived the project; JZ, ST, XQ, BL, QY and TC collected the data; and JZ, ML, ST, and XQ analyzed the data. JZ wrote the first draft of the manuscript, and all authors contributed to manuscript revision. All authors contributed to the article and approved the submitted version.
