# Supplementary material for: Response of leaf functional traits to soil nutrients in the wet and dry seasons in a subtropical forest on an island
Source: Front Plant Sci. 2023 Dec 7;14:1236607. doi: 10.3389/fpls.2023.1236607 (PMC10748499; doi:10.3389/fpls.2023.1236607)
Supplement: Supplementary file 1 [file DataSheet_1.pdf]

Supplementary Table 1 The characteristics of CWM leaf functional traits (stoichiometric characteristics) in the dry and wet seasons on Neilingding Island. Different letters within each row indicate significant differences ($P < 0.05$) in the paired *t* tests of leaf functional traits (stoichiometric characteristics) in the dry versus wet season. Values are the mean ± SD. LTC: total leaf C; LTN: total leaf N; LTP: total leaf P; LTK: total leaf K; LTCa: total leaf Ca; LTMg: total leaf Mg; N/P: the ratio of leaf N and P; C/N: the ratio of leaf C and N; C/P: the ratio of leaf C and P.

Season	LTC %	LTN %	LTP %	LTK %	LTCa %	LTMg %	N/P	C/N	C/P
Dry season	41.43±1.66[a]	2.73±0.2[a]	0.14±0.01[b]	1.38±0.12[b]	1.33±0.08[a]	0.33±0.05[b]	20.91±1.45[a]	15.82±1.25[b]	340.74±32.56[a]
Wet season	41.36±1.74[b]	2.64±0.18[b]	0.17±0.02[a]	1.56±0.17[a]	1.22±0.06[b]	0.37±0.03[a]	16.49±1.45[b]	15.97±1.25[a]	274.48±33.8[b]

Supplementary Table 2 The characteristics of CWM leaf functional traits (morphological traits) in the dry and wet seasons on Neilingding Island. Different letters within each row indicate significant differences ($P < 0.05$) in the paired *t* tests of leaf functional traits (morphological traits) in the dry versus wet season. Values are the mean ± SD. LA: leaf area; LT: leaf thickness; SLA: specific leaf area; LDMC: leaf dry matter content; SLW: specific leaf weight; LV: leaf volume; LD: leaf density; LWC: leaf water content.

Season	LA cm^2	LT cm	SLA cm^2/g	LDMC mg/g	SLW g/cm^2	LV cm^3	LD g/cm^3	LWC %
Dry season	$51.01±8.9^b$	$0.02±0.0018^a$	$152.76±11.89^b$	$373.17±30.78^a$	$0.007±5e-04^a$	$1.31±0.27^b$	$0.28±0.02^a$	$0.59±0.04^b$
Wet season	$53.3±12.73^a$	$0.02±0.0014^a$	$180.44±17.95^a$	$316.07±28.47^b$	$0.0062±6e-04^b$	$1.35±0.32^a$	$0.25±0.02^b$	$0.65±0.04^a$

Supplementary Table 3 Correlations of CWM leaf functional traits for the 15-ha forest plot in the dry season on Neilingding Island. *** $P < 0.001$; ** $P < 0.01$; * $P < 0.05$.

	LA	LT	SLA	LDMC	SLW	LV	LD	LWC	LTC	LTN	LTP	LTK	LTCa	LTMg	N/P	C/N	C/P
LA	1	0.15	0.18^{*}	-0.03	-0.02	0.86^{**}	0.16	0.19^{*}	0.17	0.27^{***}	0.37^{***}	-0.10	-0.11	0.02	-0.10	-0.11	-0.22
LT	0.15^{**}	1	-0.01	0.31^{***}	0.48^{***}	0.56^{***}	0.25^{***}	0.12	0.39^{***}	0.34^{***}	0.48^{***}	0.21^{**}	0.16^{*}	-0.17^{*}	0.06	0.20^{**}	0.06
SLA	0.18^{**}	-0.01	1	-0.22^{**}	-0.54^{***}	0.10	-0.32^{***}	0.70^{***}	0.26^{***}	0.52^{***}	0.28^{***}	0.56^{***}	0.29^{***}	0.51^{***}	0.52^{***}	-0.17^{*}	0.04
LDMC	-0.03	0.31^{***}	-0.22^{***}	1	0.68^{***}	-0.01	0.71^{***}	-0.45^{***}	0.57^{***}	0.44^{***}	0.46^{***}	-0.29^{***}	-0.07	-0.64^{***}	0.09	0.08	-0.06
SLW	-0.02	0.48^{***}	-0.54^{***}	0.68^{***}	1	0.04	0.87^{***}	-0.21^{**}	0.56^{***}	0.04	0.12	-0.23^{**}	0.13	-0.42^{***}	0.02	0.50^{***}	0.26^{***}
LV	0.86^{***}	0.56^{***}	0.10	-0.01	0.04	1	0.05	0.10	0.09	0.30^{***}	0.50^{***}	0.002	-0.12	-0.08	-0.20^{**}	-0.13	-0.28^{***}
LD	0.16^{***}	0.25^{***}	-0.32^{***}	0.71^{***}	0.87^{***}	0.05	1	-0.07	0.70^{***}	0.10	0.09	-0.25^{***}	0.18^{*}	-0.27^{***}	0.17^{*}	0.52^{***}	0.34^{***}
LWC	0.19^{***}	0.12^{*}	0.70^{***}	-0.45^{***}	-0.21^{***}	0.10	-0.07	1	0.41	0.11	-0.14	0.56^{***}	0.54^{***}	0.76^{***}	0.62^{***}	0.38^{***}	0.54^{***}
LTC	0.17^{**}	0.39^{***}	0.26^{***}	0.57^{***}	0.56^{***}	0.09	0.70^{***}	0.41^{***}	1	0.46^{***}	0.22^{**}	0.17	0.34^{***}	0.06	0.67^{***}	0.50^{***}	0.53^{***}
LTN	0.27^{***}	0.34^{***}	0.52^{***}	0.44^{***}	0.04	0.30^{***}	0.10^{*}	0.11^{*}	0.46^{***}	1	0.83^{***}	0.33^{***}	0.004	-0.23^{***}	0.36^{***}	-0.49^{***}	-0.36^{***}
LTP	0.37^{***}	0.48^{***}	0.28^{***}	0.46^{***}	0.12^{*}	0.50^{***}	0.09	-0.14^{**}	0.22^{***}	0.83^{***}	1	0.28^{***}	-0.04	-0.38	-0.16	-0.53^{***}	-0.64^{***}

LTK	-0.10	0.21***	0.56***	-0.29***	-0.23***	0.002	-0.25***	0.56***	0.17**	0.33***	0.28***	1	0.43***	0.55***	0.23***	-0.06	0.05
LTCa	-0.11	0.16*	0.29***	-0.07	0.13*	-0.12	0.18**	0.54***	0.34**	0.004	-0.04	0.43***	1	0.48***	0.27***	0.39***	0.38***
LTMg	0.02	-0.17*	0.51***	-0.64***	-0.42***	-0.08	-0.27***	0.76***	0.06	-0.23***	-0.38***	0.55***	0.48***	1	0.33***	0.30***	0.45***
N/P	-0.10	0.06	0.52***	0.09	0.02	-0.20***	0.17**	0.62***	0.67***	0.36***	-0.16**	0.23***	0.27***	0.33***	1	0.30***	0.66***
C/N	-0.11*	0.20***	-0.17**	0.08	0.50***	-0.13*	0.52***	0.38***	0.50***	-0.49***	-0.53***	-0.06	0.39***	0.30***	0.30***	1	0.87***
C/P	-0.22***	0.06	0.04	-0.06	0.26***	-0.28***	0.34***	0.54***	0.53***	-0.36***	-0.64***	0.05	0.38***	0.45***	0.66***	0.87***	1

Supplementary Table 4 Correlations of CWM leaf functional traits for the 15-ha forest plot in the wet season on Neilingding Island. *** P < 0.001; *** P < 0.01; * P < 0.05.

	LA	LT	SLA	LDMC	SLW	LV	LD	LWC	LTC	LTN	LTP	LTK	LTCa	LTMg	N/P	C/N	C/P
LA	1	0.04	0.18*	-0.02	-0.05	0.98***	-0.02	0.1	-0.04	0.36***	0.62***	0.22**	0.26***	0.12	-0.36***	-0.27***	-0.43***
LT	0.04	1	-0.16	0.32***	0.69***	0.16	0.44***	0.43***	0.68***	0.19**	-0.11	-0.12	0.40***	0.08	0.54***	0.57***	0.53***
SLA	0.18***	-0.16**	1	-0.21**	-0.64***	0.09	-0.52***	0.52***	0.07	0.68***	0.56***	0.68***	0.002	0.64***	0.02	-0.45***	-0.38***
LDMC	-0.02	0.32***	-0.21***	1	0.57***	0.01	0.66***	-0.42***	0.57***	0.26***	0.18*	-0.43***	0.36***	-0.56***	0.02	0.29***	0.04
SLW	-0.05	0.69***	-0.64***	0.57***	1	0.05	0.90***	-0.06	0.56***	-0.25***	-0.27***	-0.50***	0.41***	-0.38***	0.29***	0.80***	0.59***
LV	0.98***	0.16**	0.09	0.01	0.05	1	0.018	0.1	0.006	0.33***	0.54***	0.14	0.271***	0.06	-0.30***	-0.21**	-0.36***
LD	-0.02	0.44***	-0.52***	0.66***	0.90***	0.02	1	-0.01	0.60***	-0.20**	-0.17*	-0.46***	0.44***	-0.36***	0.22**	0.77***	0.51***
LWC	0.10*	0.43***	0.52***	-0.42***	-0.06	0.1	-0.09	1	0.39***	0.30***	0.0004	0.51***	0.18*	0.83***	0.57***	0.24***	0.38***
LTC	-0.04	0.68***	0.07	0.57***	0.56***	0.01	0.60***	0.39***	1	0.4***	0.02	-0.07	0.43***	0.11	0.59***	0.61***	0.53***
LTN	0.36***	0.19***	0.68***	0.26***	-0.25***	0.33***	-0.20***	0.30***	0.40***	1	0.69***	0.49***	0.24***	0.33**	0.11	-0.44***	-0.39***
LTP	0.62***	-0.11*	0.56***	0.18***	-0.27***	0.54***	-0.17**	0.0004	0.02	0.69***	1	0.57***	0.35***	0.20**	-0.53***	-0.50***	-0.74***

LTK	0.22***	-0.12*	0.68***	-0.43***	-0.50***	0.14**	-0.46***	0.51***	-0.07	0.45***	0.57***	1	0.23***	0.71***	-0.12	-0.37***	-0.34***
LTCa	0.26***	0.4***	0.002	3.60E+07	0.40***	0.27***	0.44***	0.18***	0.43***	0.24***	0.35***	0.23***	1	0.1	-0.07	0.27***	0.004
LTMg	0.12*	0.08	0.64***	-0.56***	-0.38***	0.06	-0.36***	0.83***	0.11***	0.33***	0.2***	0.71***	0.1*	1	0.24***	-0.09	0.04
N/P	-0.36***	0.54***	0.02	0.02	0.29***	-0.30***	0.22***	0.57***	0.59***	0.11*	-0.53***	-0.12*	-0.07	0.24***	1	0.48***	0.82***
C/N	-0.27***	0.57***	-0.45***	0.29***	0.80***	-0.21***	0.77***	0.24***	0.61***	-0.44***	-0.51***	-0.37***	0.27***	-0.09	0.48***	1	0.85***
C/P	-0.43***	0.53***	-0.38***	0.04	0.59***	-0.36***	0.51***	0.38***	0.53***	-0.39***	-0.74***	-0.34***	0.004	0.04	0.82***	0.85***	1

Supplementary Table 5 Results of standardized major axis (SMA) regression analysis for all pairwise combinations of CWM leaf functional traits in the dry and wet seasons on Neilingding Island. Slopes with different superscripts differ significantly at $P = 0.05$ between the dry and wet season. Significant results ($P < 0.05$) are shown in bold.

Trait pair (*X* and *Y*)	Season	*n*	r^2	*P*	Slope	Intercept	Slope homogeneity (*P*)	Shift in elevation (*P*)	Shift along slope (*P*)
C:N and C:P	dry	374	0.764	**< 0.001**	0.828[a]	-0.898			
	wet	374	0.735	**< 0.001**	0.632[b]	-0.336			
LA and C:N	dry	374	0.013	**0.030**	-2.028[a]	4.132			
	wet	374	0.075	**< 0.001**	-2.695[b]	4.956			
LA and C:P	dry	374	0.041	**< 0.001**	-1.680[a]	5.952	**< 0.001**	**< 0.001**	0.848
	wet	374	0.190	**< 0.001**	-1.702[a]	5.861			
LA and LD	dry	374	0.017	**0.012**	2.755	3.208			
	wet	374	0.002	0.402	-2.934	-0.071			
LA and LDMC	dry	374	0.000	0.708	-1.928	6.658			

	wet	374	0.000	0.912	2.328	-4.098			
LA and LT	dry	374	0.026	**0.002**	2.329[b]	5.441			
	wet	374	0.002	0.340	3.636[a]	7.560			
LA and LTC	dry	374	0.034	**< 0.001**	3.89	-4.588			
	wet	374	0.000	0.781	-4.871	9.589			
LA and LTCa	dry	374	0.010	0.054	-2.616	2.020			
	wet	374	0.053	**< 0.001**	4.105	1.357			
LA and LTK	dry	374	0.005	0.195	-1.858	1.961			
	wet	374	0.054	**< 0.001**	1.925	1.351			
LA and LTMg	dry	374	0.004	0.243	1.174	2.279			
	wet	374	0.023	**0.003**	2.572	2.825			
LA and LTN	dry	374	0.090	**< 0.001**	2.196[b]	0.748			
	wet	374	0.150	**< 0.001**	3.122[a]	0.404			
LA and LTP	dry	374	0.138	**< 0.001**	1.775[a]	3.243	**< 0.001**	**< 0.001**	0.213

	wet	374	0.393	**< 0.001**	1.920[a]	3.210			
LA and LV	dry	374	0.797	**< 0.001**	0.868[b]	1.607			
	wet	374	0.957	**< 0.001**	1.011[a]	1.596			
LA and LWC	dry	374	0.046	**< 0.001**	2.685[b]	2.312			
	wet	374	0.013	**0.030**	3.831[a]	2.434			
LA and N:P	dry	374	0.003	0.284	-2.286	4.717			
	wet	374	0.119	**< 0.001**	-2.361	4.586			
LA and SLA	dry	374	0.054	**< 0.001**	2.031[a]	-2.731	**< 0.001**	**< 0.001**	0.469
	wet	374	0.055	**< 0.001**	2.138[a]	-3.104			
LA and SLW	dry	374	0.002	0.420	-2.179	-3.002			
	wet	374	0.004	0.227	-2.317	-3.397			
LD and C:N	dry	374	0.292	**< 0.001**	0.736[b]	-1.429			
	wet	374	0.597	**< 0.001**	0.919[a]	-1.713			
LD and C:P	dry	374	0.141	**< 0.001**	0.610[a]	-2.090	**< 0.001**	**0.033**	0.445

	wet	374	0.292	**< 0.001**	0.580[a]	-2.022			
LD and LTC	dry	374	0.498	**< 0.001**	1.412[b]	-2.830			
	wet	374	0.372	**< 0.001**	1.660[a]	-3.293			
LD and LTCa	dry	374	0.041	**< 0.001**	0.949[b]	-0.662			
	wet	374	0.196	**< 0.001**	1.399[a]	-0.732			
LD and LTK	dry	374	0.059	**< 0.001**	-0.674[a]	-0.453	**< 0.001**	**< 0.001**	0.686
	wet	374	0.224	**< 0.001**	-0.656[a]	-0.485			
LD and LTMg	dry	374	0.067	**< 0.001**	-0.426[a]	-0.756			
	wet	374	0.122	**< 0.001**	-0.877[b]	-0.987			
LD and LTN	dry	374	0.015	**0.017**	0.797[a]	-0.893			
	wet	374	0.032	**< 0.001**	-1.064[b]	-0.162			
LD and LTP	dry	374	0.011	**0.043**	0.644[a]	0.013	**< 0.001**	0.252	0.831
	wet	374	0.027	**0.001**	-0.655[a]	-1.118			
LD and LWC	dry	374	0.001	0.463	-0.975	-0.768			

	wet	374	0.003	0.259	-1.306	-0.854			
LD and N:P	dry	374	0.045	**< 0.001**	0.830[a]	-1.641	**< 0.001**	**< 0.001**	0.667
	wet	374	0.067	**< 0.001**	0.805[a]	-1.587			
LDMC and C:N	dry	374	0.007	0.102	1.052	1.310			
	wet	374	0.078	**< 0.001**	1.158	1.106			
LDMC and C:P	dry	374	0.001	0.564	-0.871	4.775			
	wet	374	0.005	0.169	0.731	0.717			
LDMC and LD	dry	374	0.495	**< 0.001**	1.429[a]	3.352			
	wet	374	0.418	**< 0.001**	1.260[b]	3.266			
LDMC and LTC	dry	374	0.323	**< 0.001**	2.018[a]	-0.692	**< 0.001**	**< 0.001**	0.544
	wet	374	0.322	**< 0.001**	2.093[a]	-0.884			
LDMC and LTCa	dry	374	0.004	0.242	-1.357	2.735			
	wet	374	0.126	**< 0.001**	1.764	2.344			
LDMC and LTK	dry	374	0.081	**< 0.001**	-0.964[b]	2.705			

	wet	374	0.188	**< 0.001**	-0.827[a]	2.655			
LDMC and LTMg	dry	374	0.409	**< 0.001**	-0.609[a]	2.271			
	wet	374	0.300	**< 0.001**	-1.105[b]	2.022			
LDMC and LTN	dry	374	0.218	**< 0.001**	1.139[b]	2.076			
	wet	374	0.083	**< 0.001**	1.341[a]	1.934			
LDMC and LTP	dry	374	0.239	**< 0.001**	0.921[a]	3.370	**< 0.001**	**< 0.001**	0.105
	wet	374	0.052	**< 0.001**	0.825[a]	3.140			
LDMC and LV	dry	374	0.000	0.786	0.45	2.521			
	wet	374	0.002	0.438	0.434	2.446			
LDMC and LWC	dry	374	0.175	**< 0.001**	-1.393[a]	2.254			
	wet	374	0.152	**< 0.001**	-1.646[b]	2.190			
LDMC and N:P	dry	374	0.013	**0.026**	1.185	1.007			
	wet	374	0.003	0.275	1.014	1.265			
LDMC and SLW	dry	374	0.458	**< 0.001**	1.130[a]	5.010			

	wet	374	0.313	**< 0.001**	0.996[b]	4.695			
LT and C:N	dry	374	0.054	**< 0.001**	0.871[a]	-2.649			
	wet	374	0.350	**< 0.001**	0.741[b]	-2.498			
LT and C:P	dry	374	0.010	0.051	0.721	-3.431			
	wet	374	0.314	**< 0.001**	0.468	-2.747			
LT and LD	dry	374	0.080	**< 0.001**	1.183[a]	-0.959			
	wet	374	0.211	**< 0.001**	0.807[b]	-1.116			
LT and LDMC	dry	374	0.118	**< 0.001**	0.828[a]	-3.734			
	wet	374	0.113	**< 0.001**	0.640[b]	-3.207			
LT and LTC	dry	374	0.190	**< 0.001**	1.671[a]	-4.307			
	wet	374	0.488	**< 0.001**	1.340[b]	-3.773			
LT and LTCa	dry	374	0.035	**< 0.001**	1.123[a]	-1.742	**< 0.001**	**< 0.001**	0.941
	wet	374	0.173	**< 0.001**	1.129[a]	-1.706			
LT and LTK	dry	374	0.039	**< 0.001**	0.798[a]	-1.717			

	wet	374	0.011	**0.045**	-0.529[b]	-1.507			
LT and LTMg	dry	374	0.027	**0.001**	-0.504	-1.854			
	wet	374	0.010	0.055	0.707	-1.303			
LT and LTN	dry	374	0.124	**< 0.001**	0.943[a]	-2.016	**< 0.001**	**< 0.001**	0.181
	wet	374	0.044	**< 0.001**	0.859[a]	-1.968			
LT and LTP	dry	374	0.205	**< 0.001**	0.762[a]	-0.944			
	wet	374	0.011	**0.038**	-0.528[b]	-2.018			
LT and LV	dry	374	0.264	**< 0.001**	0.373[a]	-1.647			
	wet	374	0.038	**< 0.001**	0.278[b]	-1.640			
LT and LWC	dry	374	0.019	**0.007**	1.153[a]	-1.344	**< 0.001**	**< 0.001**	0.193
	wet	374	0.199	**< 0.001**	1.054[a]	-1.410			
LT and N:P	dry	374	0.011	**0.046**	0.982[a]	-2.901			
	wet	374	0.331	**< 0.001**	0.649[b]	-2.397			
LT and SLA	dry	374	0.000	0.995	-0.872	0.298			

	wet	374	0.015	**0.016**	-0.588	-0.281			
LT and SLW	dry	374	0.274	**< 0.001**	0.936[a]	0.414			
	wet	374	0.502	**< 0.001**	0.637[b]	-0.201			
LTC and C:N	dry	374	0.268	**< 0.001**	0.521[a]	0.992	**0.579**	**0.016**	< 0.001
	wet	374	0.387	**< 0.001**	0.553[a]	0.951			
LTC and C:P	dry	374	0.317	**< 0.001**	0.432[a]	0.524			
	wet	374	0.314	**< 0.001**	0.349[b]	0.765			
LTC and LTCa	dry	374	0.125	**< 0.001**	0.672[b]	1.535			
	wet	374	0.190	**< 0.001**	0.843[a]	1.542			
LTC and LTK	dry	374	0.031	**0.001**	0.478	1.550			
	wet	374	0.003	0.298	-0.395	1.691			
LTC and LTMg	dry	374	0.005	0.164	0.302	1.765			
	wet	374	0.014	**0.021**	0.528	1.844			
LTC and LTN	dry	374	0.219	**< 0.001**	0.565[a]	1.372	**< 0.001**	**< 0.001**	< 0.001

	wet	374	0.170	**< 0.001**	0.641[a]	1.347
LTC and LTP	dry	374	0.049	**< 0.001**	0.456	2.013
	wet	374	0.001	0.503	0.394	1.923
LTC and N:P	dry	374	0.477	**< 0.001**	0.588[a]	0.842
	wet	374	0.382	**< 0.001**	0.485[b]	1.027
LTCa and C:N	dry	374	0.155	**< 0.001**	0.775[a]	-0.807
	wet	374	0.079	**< 0.001**	0.657[b]	-0.702
LTCa and C:P	dry	374	0.150	**< 0.001**	0.642	-1.504
	wet	374	0.001	0.635	0.415	-0.922
LTCa and LTMg	dry	374	0.225	**< 0.001**	0.449[b]	0.342
	wet	374	0.010	**0.050**	0.627[a]	0.358
LTCa and N:P	dry	374	0.089	**< 0.001**	0.874	-1.031
	wet	374	0.002	0.421	-0.575	0.787
LTK and C:N	dry	374	0.004	0.226	-1.092	1.447

	wet	374	0.141	**< 0.001**	-1.4	1.873
LTK and C:P	dry	374	0.002	0.409	0.904	-2.149
	wet	374	0.120	**< 0.001**	-0.884	2.343
LTK and LTCa	dry	374	0.181	**< 0.001**	1.408[b]	-0.032
	wet	374	0.044	**< 0.001**	2.132[a]	0.003
LTK and LTMg	dry	374	0.296	**< 0.001**	0.632[b]	0.450
	wet	374	0.514	**< 0.001**	1.336[a]	0.766
LTK andN:P	dry	374	0.061	**< 0.001**	1.23	-1.484
	wet	374	0.010	0.053	-1.226	1.681
LTMg and C:N	dry	374	0.086	**< 0.001**	1.728	-2.561
	wet	374	0.007	0.106	-1.048	0.829
LTMg and C:P	dry	374	0.176	**< 0.001**	1.431	-4.112
	wet	374	0.000	0.716	0.662	-2.043
LTMg and N:P	dry	374	0.116	**< 0.001**	1.947[a]	-3.060

	wet	374	0.058	**< 0.001**	0.918^{b}	-1.547			
LTN and C:N	dry	374	0.221	**< 0.001**	-0.923^{a}	1.541	**< 0.001**	**< 0.001**	< 0.001
	wet	374	0.173	**< 0.001**	-0.863^{a}	1.458			
LTN and C:P	dry	374	0.109	**< 0.001**	-0.765^{b}	2.370			
	wet	374	0.128	**< 0.001**	-0.545^{a}	1.748			
LTN and LTCa	dry	374	0.000	0.748	1.191	0.290			
	wet	374	0.063	**< 0.001**	1.315	0.305			
LTN and LTK	dry	374	0.119	**< 0.001**	0.846^{a}	0.317			
	wet	374	0.260	**< 0.001**	0.617^{b}	0.303			
LTN and LTMg	dry	374	0.042	**< 0.001**	-0.535^{b}	0.172			
	wet	374	0.123	**< 0.001**	0.824^{a}	0.775			
LTN and LTP	dry	374	0.706	**< 0.001**	0.808^{a}	1.136			
	wet	374	0.503	**< 0.001**	0.615^{b}	0.899			
LTN and N:P	dry	374	0.134	**< 0.001**	1.041^{a}	-0.938			

	wet	374	0.016	**0.014**	0.756[b]	-0.499			
LTP and C:N	dry	374	0.275	**< 0.001**	-1.142[a]	0.501			
	wet	374	0.259	**< 0.001**	-1.403[b]	0.909			
LTP and C:P	dry	374	0.392	**< 0.001**	-0.946[a]	1.526	**< 0.001**	0.367	0.225
	wet	374	0.535	**< 0.001**	-0.886[a]	1.381			
LTP and LTCa	dry	374	0.002	0.391	-1.473	-0.689			
	wet	374	0.115	**< 0.001**	2.138	-0.965			
LTP and LTK	dry	374	0.075	**< 0.001**	1.046[a]	-1.014	**< 0.001**	**< 0.001**	**< 0.001**
	wet	374	0.283	**< 0.001**	1.002[a]	-0.968			
LTP and LTMg	dry	374	0.143	**< 0.001**	-0.661[b]	-1.193			
	wet	374	0.035	**< 0.001**	1.339[a]	-0.201			
LTP and N:P	dry	374	0.020	**0.006**	-1.287[a]	0.831	**< 0.001**	**< 0.001**	0.497
	wet	374	0.269	**< 0.001**	-1.229[a]	0.716			
LV and C:N	dry	374	0.015	**0.017**	-2.337[a]	2.908	0.989	**0.013**	0.069

	wet	374	0.041	**< 0.001**	-2.665[a]	3.323
LV and C:P	dry	374	0.065	**< 0.001**	-1.935[b]	5.006
	wet	374	0.123	**< 0.001**	-1.684[a]	4.219
LV and LD	dry	374	0.003	0.321	3.174	1.844
	wet	374	0.000	0.960	2.901	1.887
LV and LTC	dry	374	0.017	**0.013**	4.482	-7.139
	wet	374	0.001	0.499	4.818	-7.667
LV and LTCa	dry	374	0.012	**0.036**	-3.014[b]	0.475
	wet	374	0.064	**< 0.001**	4.06[a]	-0.237
LV and LTK	dry	374	0.000	0.994	2.141	-0.189
	wet	374	0.023	**0.003**	1.904	-0.243
LV and LTMg	dry	374	0.003	0.296	-1.353	-0.556
	wet	374	0.008	0.080	2.543	1.215
LV and LTN	dry	374	0.106	**< 0.001**	2.531[b]	-0.990

	wet	374	0.129	**< 0.001**	3.087[a]	-1.179			
LV and LTP	dry	374	0.233	**< 0.001**	2.046[a]	1.884	**< 0.001**	**< 0.001**	0.239
	wet	374	0.292	**< 0.001**	1.899[a]	1.596			
LV and LWC	dry	374	0.015	**0.017**	3.094[b]	0.812			
	wet	374	0.012	**0.037**	3.789[a]	0.828			
LV and N:P	dry	374	0.022	**0.004**	-2.633[a]	3.583	**< 0.001**	**< 0.001**	0.093
	wet	374	0.074	**< 0.001**	-2.335[a]	2.957			
LWC and C:N	dry	374	0.143	**< 0.001**	0.755[a]	-1.132	**< 0.001**	**< 0.001**	0.305
	wet	374	0.064	**< 0.001**	0.703[a]	-1.033			
LWC and C:P	dry	374	0.280	**< 0.001**	0.626[a]	-1.810			
	wet	374	0.138	**< 0.001**	0.444[b]	-1.269			
LWC and LTC	dry	374	0.185	**< 0.001**	1.449[a]	-2.570			
	wet	374	0.172	**< 0.001**	1.272[b]	-2.242			
LWC and LTCa	dry	374	0.300	**< 0.001**	0.974[a]	-0.346	0.086	**< 0.001**	**< 0.001**

	wet	374	0.040	**< 0.001**	1.071[a]	-0.281			
LWC and LTK	dry	374	0.313	**< 0.001**	0.692[a]	-0.324			
	wet	374	0.281	**< 0.001**	0.502[b]	-0.283			
LWC and LTMg	dry	374	0.592	**< 0.001**	0.437[b]	-0.012			
	wet	374	0.691	**< 0.001**	0.671[a]	0.102			
LWC and LTN	dry	374	0.018	**0.010**	0.818[a]	-0.583	**< 0.001**	**< 0.001**	**< 0.001**
	wet	374	0.104	**< 0.001**	0.815[a]	-0.530			
LWC and LTP	dry	374	0.018	**0.010**	-0.661	-0.801			
	wet	374	0.000	0.848	-0.501	-0.577			
LWC and N:P	dry	374	0.400	**< 0.001**	0.851[a]	-1.350			
	wet	374	0.330	**< 0.001**	0.616[b]	-0.936			
N:P and C:N	dry	374	0.104	**< 0.001**	0.887[b]	0.256			
	wet	374	0.255	**< 0.001**	1.142[a]	-0.157			
N:P and C:P	dry	374	0.461	**< 0.001**	0.735[a]	-0.540	**< 0.001**	**< 0.001**	0.693

	wet	374	0.680	**< 0.001**	0.721[a]	-0.540			
SLA and C:N	dry	374	0.023	**0.003**	-0.999[a]	3.379			
	wet	374	0.189	**< 0.001**	-1.260[b]	3.769			
SLA and C:P	dry	374	0.003	0.265	0.827	0.090			
	wet	374	0.132	**< 0.001**	-0.796	4.193			
SLA and LD	dry	374	0.088	**< 0.001**	-1.356[a]	1.441	**< 0.001**	**0.001**	0.866
	wet	374	0.263	**< 0.001**	-1.372[a]	1.418			
SLA and LDMC	dry	374	0.039	**< 0.001**	-0.949[a]	4.623	**< 0.001**	0.647	0.058
	wet	374	0.037	**< 0.001**	-1.088[a]	4.973			
SLA and LTC	dry	374	0.080	**< 0.001**	1.916	-0.915			
	wet	374	0.009	0.066	2.278	-1.427			
SLA and LTCa	dry	374	0.091	**< 0.001**	1.288	2.026			
	wet	374	0.000	0.827	1.92	2.086			
SLA and LTK	dry	374	0.309	**< 0.001**	0.915[a]	2.055	**< 0.001**	**< 0.001**	**< 0.001**

	wet	374	0.485	**< 0.001**	0.900[a]	2.083			
SLA and LTMg	dry	374	0.271	**< 0.001**	0.578[b]	2.467			
	wet	374	0.426	**< 0.001**	1.203[a]	2.772			
SLA and LTN	dry	374	0.275	**< 0.001**	1.082[b]	1.713			
	wet	374	0.493	**< 0.001**	1.46[a]	1.641			
SLA and LTP	dry	374	0.075	**< 0.001**	0.874[a]	2.941	**< 0.001**	**0.037**	**< 0.001**
	wet	374	0.305	**< 0.001**	0.898[a]	2.953			
SLA and LV	dry	374	0.019	**0.008**	0.427[a]	2.136	**< 0.001**	**< 0.001**	**< 0.001**
	wet	374	0.016	**0.015**	0.473[a]	2.198			
SLA and LWC	dry	374	0.503	**< 0.001**	1.322[b]	2.483			
	wet	374	0.292	**< 0.001**	1.792[a]	2.590			
SLA and N:P	dry	374	0.289	**< 0.001**	1.125	0.698			
	wet	374	0.002	0.383	1.104	0.912			
SLA and SLW	dry	374	0.275	**< 0.001**	-1.073[a]	-0.134	**< 0.001**	**< 0.001**	**< 0.001**

	wet	374	0.389	**< 0.001**	-1.084^{a}	-0.137			
SLW and C:N	dry	374	0.256	**< 0.001**	0.931^{b}	-3.273			
	wet	374	0.641	**< 0.001**	1.163^{a}	-3.605			
SLW and C:P	dry	374	0.088	**< 0.001**	0.771^{a}	-4.109	**< 0.001**	**< 0.001**	**< 0.001**
	wet	374	0.385	**< 0.001**	0.735^{a}	-3.996			
SLW and LD	dry	374	0.752	**< 0.001**	1.264^{a}	-1.467	**< 0.001**	**< 0.001**	**< 0.001**
	wet	374	0.802	**< 0.001**	1.266^{a}	-1.436			
SLW and LTC	dry	374	0.327	**< 0.001**	1.785^{b}	-5.044			
	wet	374	0.326	**< 0.001**	2.102^{a}	-5.604			
SLW and LTCa	dry	374	0.020	**0.006**	1.200^{b}	-2.304			
	wet	374	0.164	**< 0.001**	1.771^{a}	-2.362			
SLW and LTK	dry	374	0.052	**< 0.001**	-0.852^{a}	-2.040	**< 0.001**	0.067	**< 0.001**
	wet	374	0.253	**< 0.001**	-0.831^{a}	-2.049			
SLW and LTMg	dry	374	0.171	**< 0.001**	-0.539^{a}	-2.423			

	wet	374	0.133	**< 0.001**	-1.110[b]	-2.685			
SLW and LTN	dry	374	0.004	0.210	1.008	-2.596			
	wet	374	0.054	**< 0.001**	-1.347	-1.641			
SLW and LTP	dry	374	0.017	**0.013**	0.815[a]	-1.451	**< 0.001**	**< 0.001**	**< 0.001**
	wet	374	0.073	**< 0.001**	-0.829[a]	-2.852			
SLW and LV	dry	374	0.002	0.373	0.398	-2.202			
	wet	374	0.003	0.314	0.436	-2.259			
SLW and LWC	dry	374	0.033	**< 0.001**	-1.232	-2.438			
	wet	374	0.001	0.474	-1.653	-2.516			
SLW and N:P	dry	374	0.003	0.292	1.049	-3.542			
	wet	374	0.110	**< 0.001**	1.019	-3.445			

Supplementary Table 6 Principal component contribution and leaf initial factor rotation component matrix in the dry season.

Index factor	Component 1	Component 2	Component 3	Component 4	Component 5	Common factor variance
LA	-0.012	0.098	0.132	-0.149	0.936	0.925
LT	0.525	0.217	-0.146	0.477	0.392	0.725
SLA	-0.391	0.322	0.704	0.364	0.122	0.899
LDMC	0.832	0.382	0.061	-0.260	-0.123	0.925
SLW	0.946	-0.117	-0.159	0.023	0.011	0.935
LV	0.063	0.207	-0.112	0.068	0.952	0.970
LD	0.892	-0.154	0.130	-0.092	0.088	0.852
LWC	-0.194	-0.272	0.652	0.588	0.235	0.937
LTC	0.703	0.015	0.641	0.199	0.115	0.959
LTN	0.197	0.850	0.430	0.093	0.159	0.980
LTP	0.223	0.890	-0.049	0.165	0.321	0.974
LTK	-0.222	0.260	0.213	0.813	-0.066	0.829
LTCa	0.155	-0.187	0.187	0.742	-0.111	0.656
LTMg	-0.460	-0.433	0.384	0.558	0.064	0.862
N/P	0.154	-0.092	0.905	0.127	-0.145	0.889
C/N	0.483	-0.780	0.172	0.252	0.004	0.935
C/P	0.291	-0.741	0.491	0.218	-0.142	0.943
Characteristic value	4.462	4.146	3.632	1.763	1.194	
Contribution rate (%)	26.246	24.387	21.366	10.370	7.021	
Contribution rate (%)	26.246	50.633	71.999	82.369	89.390	

Supplementary Table 7 Principal component contribution and leaf initial factor rotation component matrix in the wet season.

Index factor	Component 1	Component 2	Component 3	Component 4	Common factor variance
LA	-0.014	0.099	0.145	0.936	0.906
LT	0.829	0.172	-0.004	0.075	0.722
SLA	-0.238	0.543	0.720	-0.016	0.870
LDMC	0.556	-0.712	0.382	-0.030	0.963
SLW	0.840	-0.347	-0.317	0.094	0.936
LV	0.061	0.077	0.086	0.936	0.893
LD	0.800	-0.407	-0.191	0.076	0.848
LWC	0.345	0.912	0.100	0.001	0.961
LTC	0.886	0.070	0.323	-0.128	0.910
LTN	0.113	0.195	0.902	0.160	0.889
LTP	-0.157	-0.022	0.745	0.578	0.914
LTK	-0.236	0.629	0.492	0.190	0.729
LTCa	0.533	-0.027	0.273	0.405	0.523
LTMg	-0.023	0.903	0.221	0.042	0.867
N/P	0.586	0.430	-0.089	-0.513	0.800
C/N	0.803	0.001	-0.453	-0.174	0.880
C/P	0.680	0.234	-0.503	-0.438	0.962
Characteristic value	5.947	3.893	3.237	1.498	
Contribution rate (%)	34.980	22.902	19.040	8.809	
Contribution rate (%)	34.980	57.882	76.922	85.731	

Supplementary Table 8 List of the 79 plant species in the 15-ha subtropical forest plot on Neilingding Island.

Species	Genus	Family	Number of trees sampled (dry season)	Number of trees sampled (wet season)
Glochidion lanceolarium	Glochidion	Phyllanthaceae	1	1
Flueggea virosa	Flueggea	Euphorbiaceae	1	1
Artocarpus hypargyreus	Artocarpus	Moraceae	9	8
Tarenna mollissima	Tarenna	Rubiaceae	1	1
Mallotus paniculatus	Mallotus	Euphorbiaceae	9	11
Claoxylon indicum	Claoxylon	Euphorbiaceae	6	6
Scolopia chinensis	Scolopia	Salicaceae	4	4
Litsea rotundifolia	Litsea	Lauraceae	10	10
Litsea verticillata	Litsea	Lauraceae	10	9
Ailanthus fordii	Ailanthus	Simaroubaceae	4	2
Phoenix loureiroi	Phoenix	Arecaceae	8	8
Mallotus philippensis	Mallotus	Euphorbiaceae	6	6
Ficus hirta	Ficus	Moraceae	2	3
Clerodendrum cyrtophyllum	Clerodendrum	Verbenaceae	2	1
Laurocerasus zippeliana	Laurocerasus	Rosaceae	2	2
Glochidion macrophyllum	Glochidion	Euphorbiaceae	2	2
Ficus hispida	Ficus	Moraceae	14	15
Schefflera octophylla	Schefflera	Araliaceae	10	9
Psidium guajava	Psidium	Myrtaceae	2	1
Pterospermum heterophyllum	Pterospermum	Malvaceae	11	9
Koelreuteria bipinnata	Koelreuteria	Sapindaceae	6	6
Citrus reticulata	Citrus	Rutaceae	2	10

Breynia fruticosa	Breynia	Euphorbiaceae	6	5
Thevetia peruviana	Thevetia	Apocynaceae	1	1
Aralia decaisneana	Aralia	Araliaceae	5	5
Cratoxylum cochinchinense	Cratoxylum	Hypericaceae	5	5
Maesa perlarius	Maesa	Primulaceae	4	3
Tarenna attenuata	Tarenna	Rubiaceae	20	19
Sterculia lanceolata	Sterculia	Sterculiaceae	12	12
Litsea monopetala	Litsea	Lauraceae	7	8
Desmos chinensis	Desmos	Annonaceae	10	10
Psychotria asiatica	Psychotria	Rubiaceae	13	14
Atalantia buxifolia	Atalantia	Rutaceae	5	5
Zanthoxylum avicennae	Zanthoxylum	Rutaceae	9	10
Litchi chinensis	Litchi	Sapindaceae	7	7
Melia azedarach	Melia	Meliaceae	2	3
Archidendron lucidum	Archidendron	Leguminosae	8	8
Maesa salicifolia	Maesa	Primulaceae	4	5
Dimocarpus longan	Dimocarpus	Sapindaceae	10	11
Callicarpa nudiflora	Callicarpa	Verbenaceae	9	8
Alangium kurzii	Alangium	Alangiaceae	6	8
Antirhea chinensis	Antirhea	Rubiaceae	1	1
Oroxylum indicum	Oroxylum	Bignoniaceae	3	3
Citrus limon	Citrus	Rutaceae	1	2
Strychnos angustiflora	Strychnos	Loganiaceae	3	3
Microcos paniculata	Microcos	Tiliaceae	20	24
Celtis sinensis	Celtis	Ulmaceae	2	2
Ficus variegata	Ficus	Moraceae	3	3

Bischofia javanica	Bischofia	Euphorbiaceae	8	8
Casearia glomerata	Casearia	Salicaceae	12	12
Sageretia thea	Sageretia	Rhamnaceae	5	6
Ficus microcarpa	Ficus	Moraceae	6	7
Cansjera rheedei	Cansjera	Opiliaceae	2	2
Trema tomentosa	Trema	Ulmaceae	3	10
Vitex quinata	Vitex	Verbenaceae	10	3
Syzygium levinei	Syzygium	Myrtaceae	16	16
Triadica cochinchinensis	Triadica	Euphorbiaceae	1	1
Glycosmis pentaphylla	Glycosmis	Rutaceae	10	11
Viburnum odoratissimum	Viburnum	Caprifoliaceae	3	3
Euonymus laxiflorus	Euonymus	Celastraceae	1	1
Itea chinensis	Itea	Saxifragaceae	5	6
Ficus fistulosa	Ficus	Moraceae	8	7
Glochidion puberum	Glochidion	Euphorbiaceae	4	3
Acacia confusa	Acacia	Fabaceae	10	10
Dallergiahancai Benth	Dalbergia	Fabaceae	1	1
Ilex rotunda	Ilex	Aquifoliaceae	2	2
Bridelia tomentosa	Bridelia	Euphorbiaceae	12	12
Euonymus alatus	Euonymus	Celastraceae	1	1
Triadica sebifera	Triadica	Euphorbiaceae	5	5
Antidesma bunius	Antidesma	Euphorbiaceae	6	6
Fissistigma uonicum	Fissistigma	Annonaceae	7	7
Emmenopterys henryi	Emmenopterys	Rubiaceae	2	2
Glycosmis parviflora	Glycosmis	Rutaceae	1	1
Macaranga tanarius	Macaranga	Euphorbiaceae	11	13

Brucea javanica	Brucea	Simaroubaceae	5	5
Strophanthus divaricatus	Strophanthus	Apocynaceae	3	3
Aporosa dioica	Aporosa	Euphorbiaceae	10	10
Trigonostemon wui	Trigonostemon	Euphorbiaceae	4	2
Cinnamomum camphora	Cinnamomum	Lauraceae	2	2

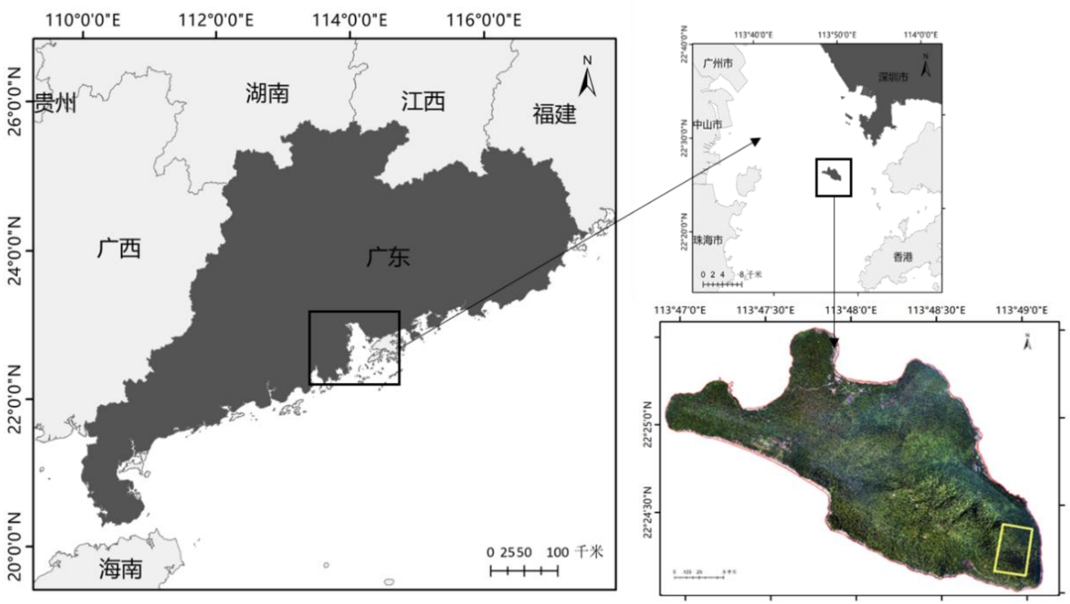

Fig. S1. Location of the 15-ha plot on Neilingding Island in southern China (the rectangle shows the plot location and dimensions).

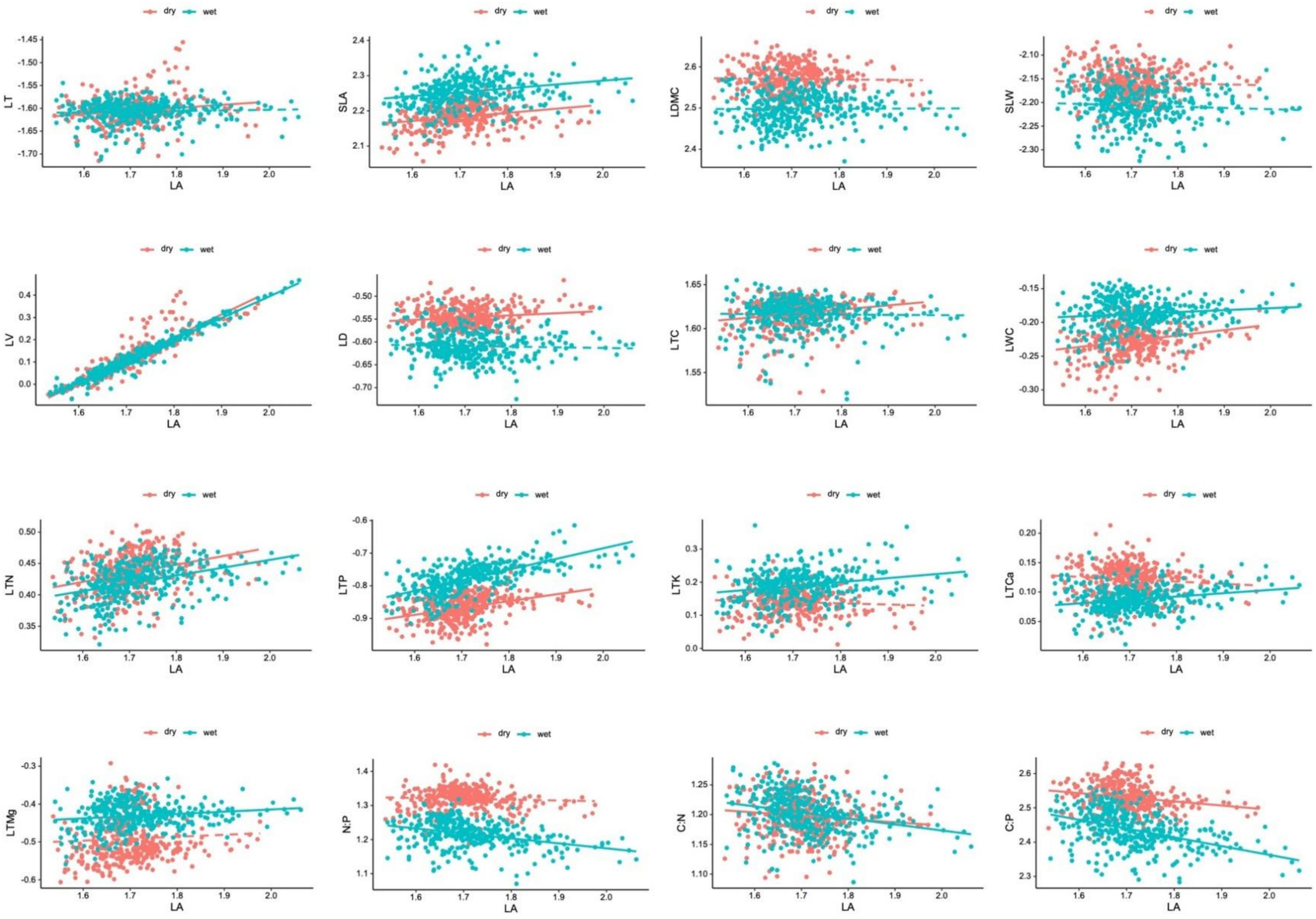
dry
wet
LA
LT
SLA
LDMC
SLW
LV
LD
LTC
LWC
LTN
LTP
LTK
LTCa
LTMg
N:P
C:N
C:P

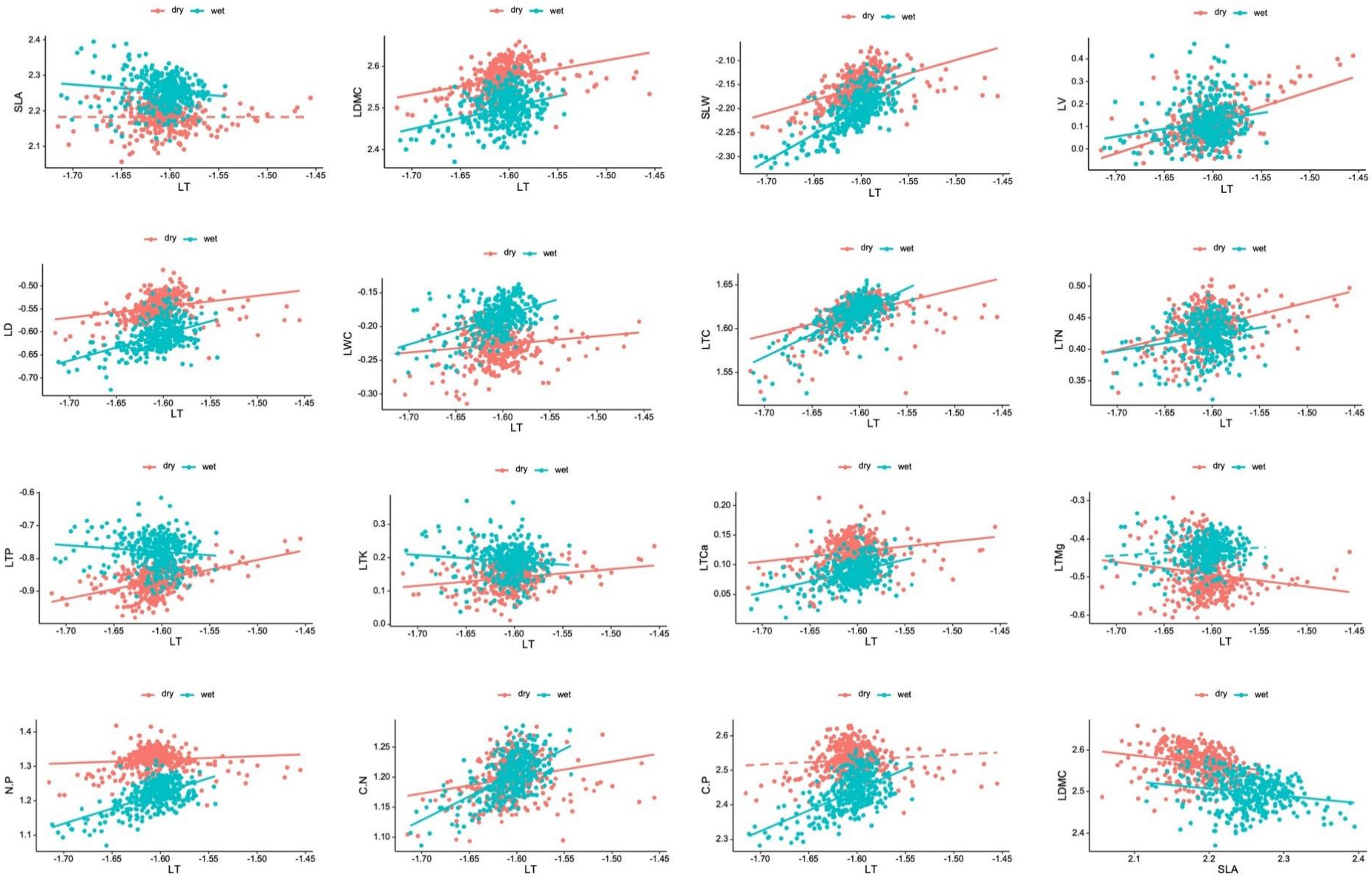
dry
wet
SLA
LDMC
SLW
LV
LD
LWC
LTC
LTN
LTP
LTK
LTCa
LTMg
N.P
C.N
C.P
LT

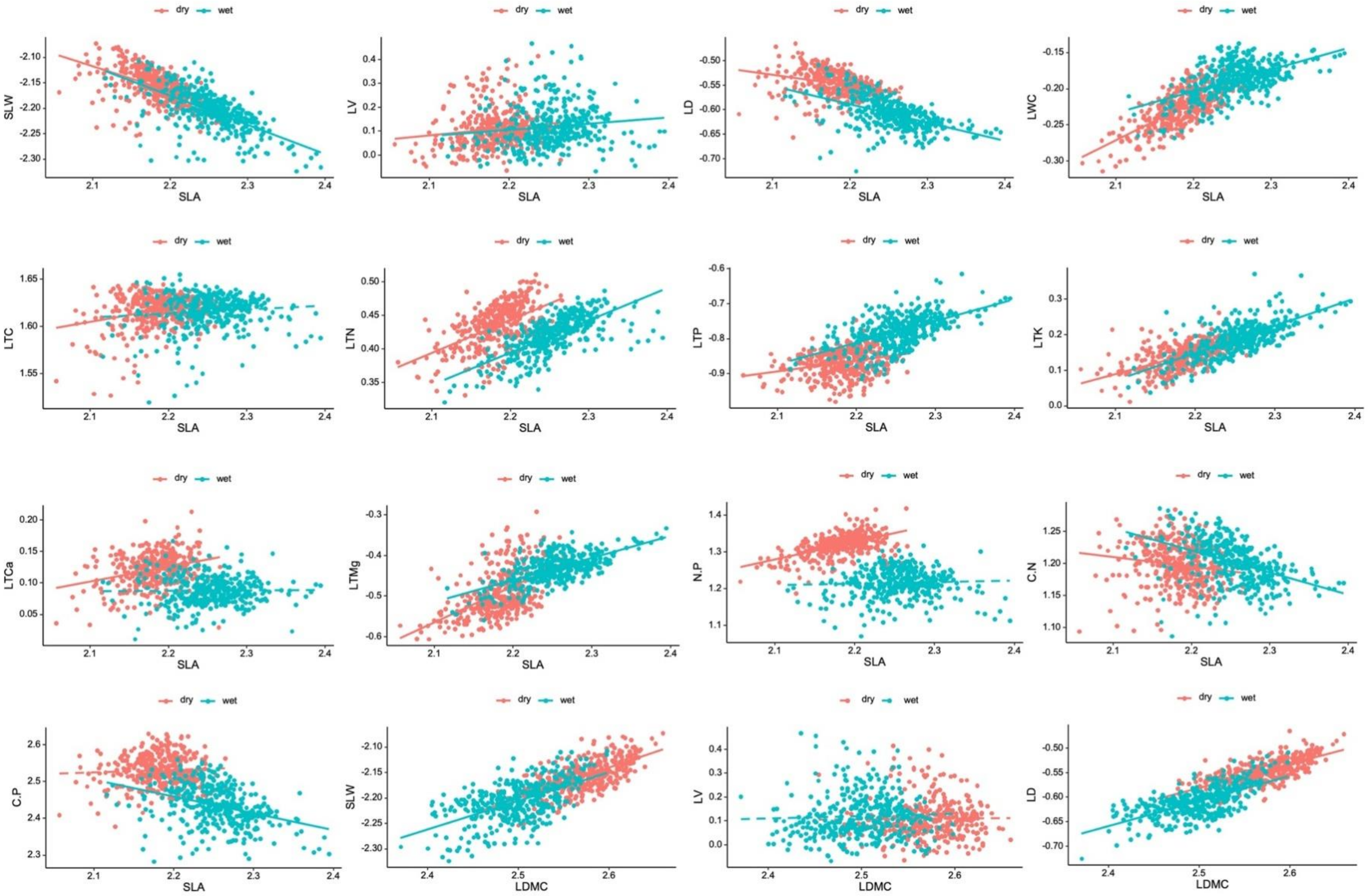
dry wet
SLW
SLA
LV
SLA
LD
SLA
LWC
SLA
LTC
SLA
LTN
SLA
LTP
SLA
LTK
SLA
LTCa
SLA
LTMg
SLA
N.P
SLA
C.N
SLA
C.P
SLA
SLW
LDMC
LV
LDMC
LD
LDMC

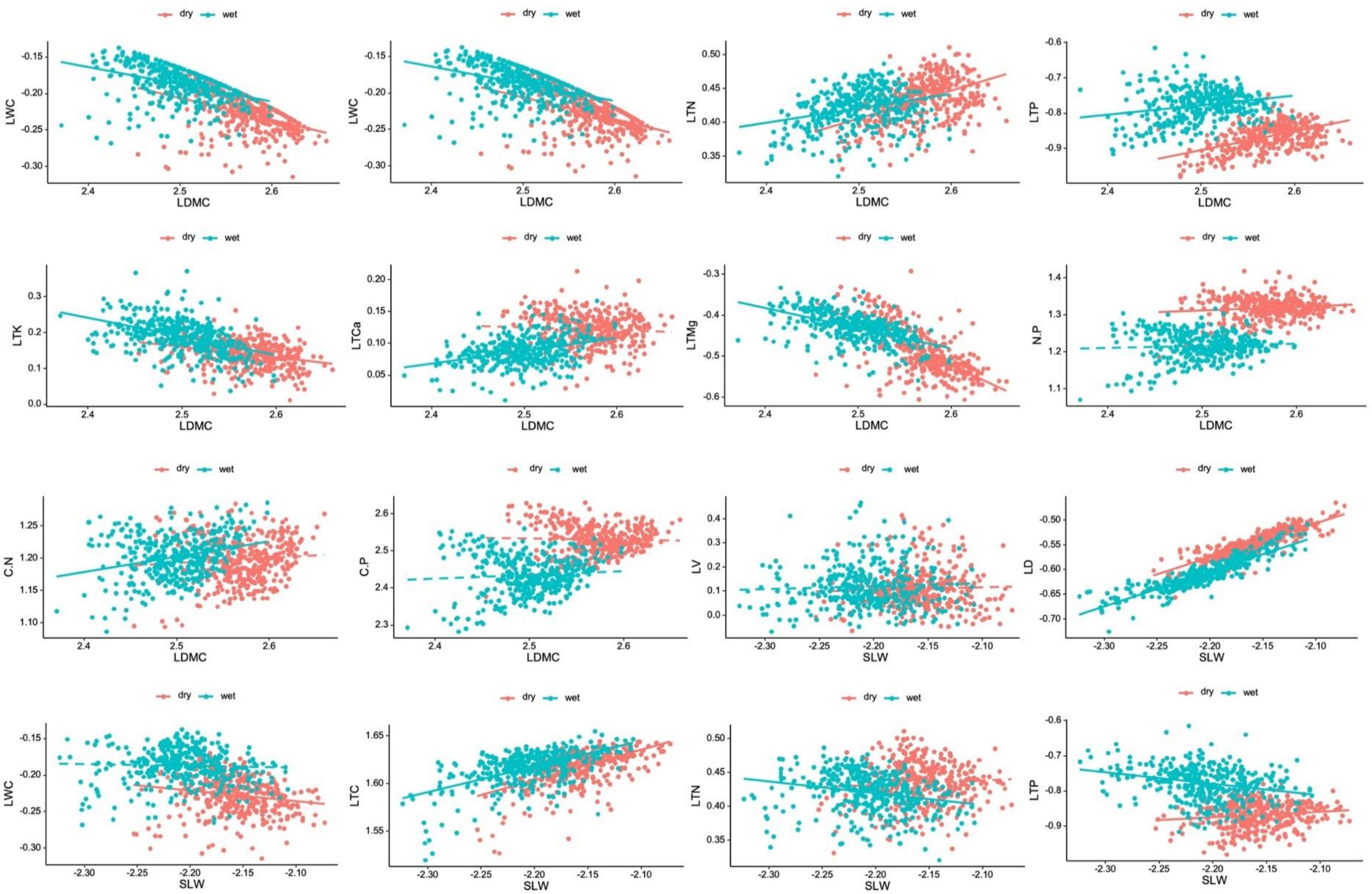
dry
wet
LWC
LDMC
LWC
LDMC
LTN
LDMC
LTP
LDMC
LTK
LDMC
LTCa
LDMC
LTMg
LDMC
N.P
LDMC
C.N
LDMC
C.P
LDMC
LV
SLW
LD
SLW
LWC
SLW
LTC
SLW
LTN
SLW
LTP
SLW

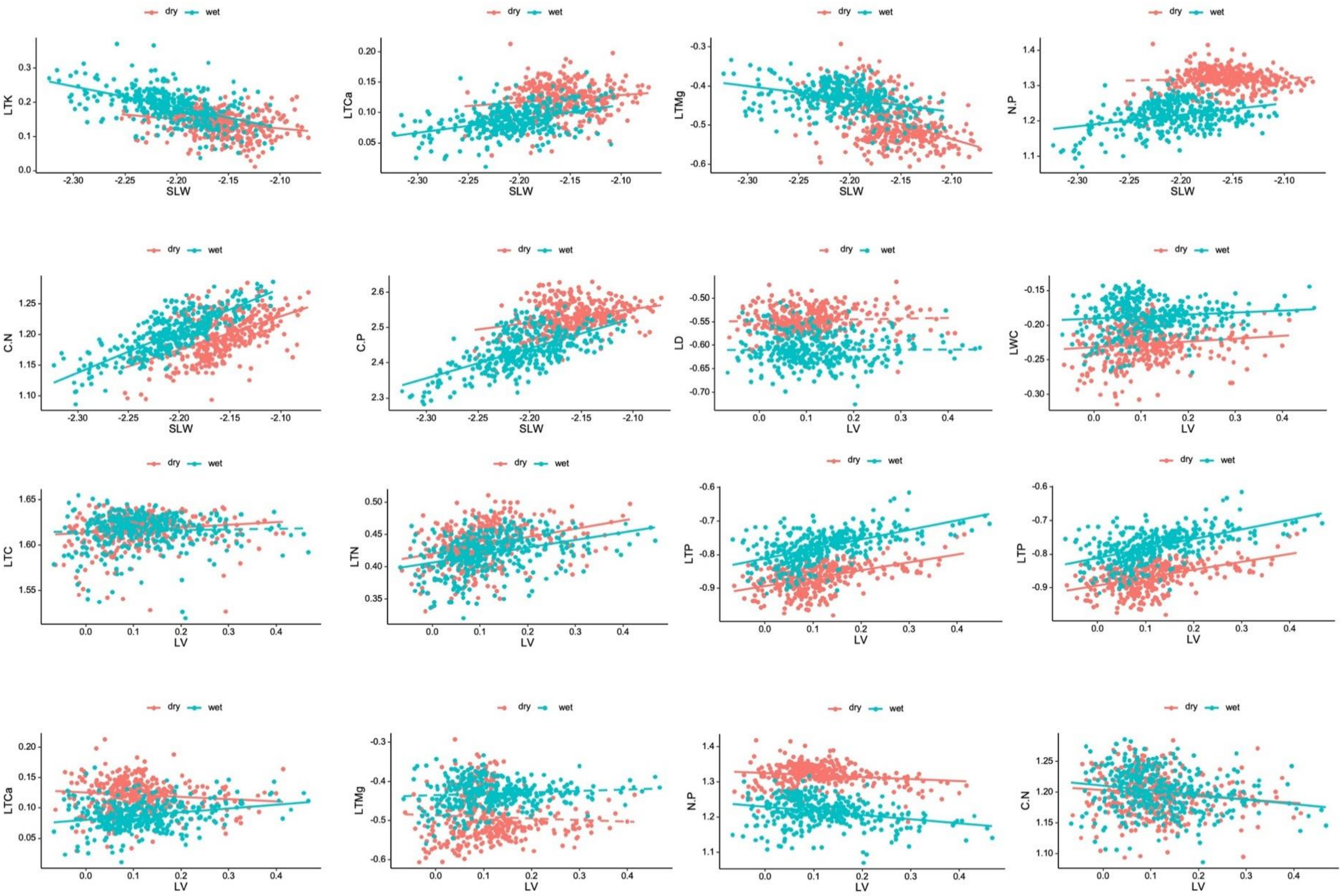
dry wet
LTK
SLW
LTCa
SLW
LTMg
SLW
N.P
SLW
C.N
SLW
C.P
SLW
LD
LV
LWC
LV
LTC
LV
LTN
LV
LTP
LV
LTP
LV
LTCa
LV
LTMg
LV
N.P
LV
C.N
LV

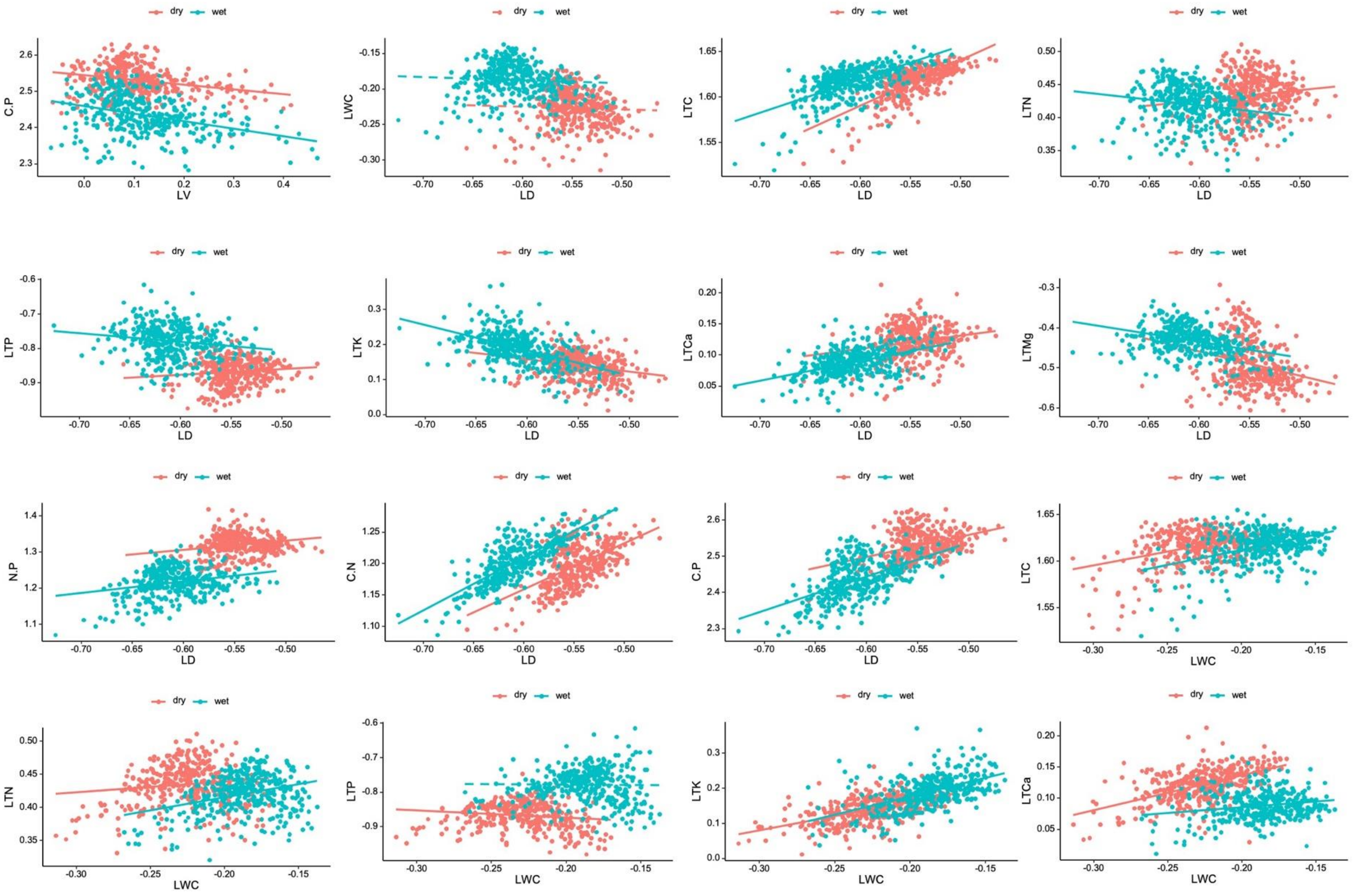
dry wet
C.P
LV
LWC
LD
LTC
LTN
LTP
LTK
LTCa
LTMg
N.P
C.N

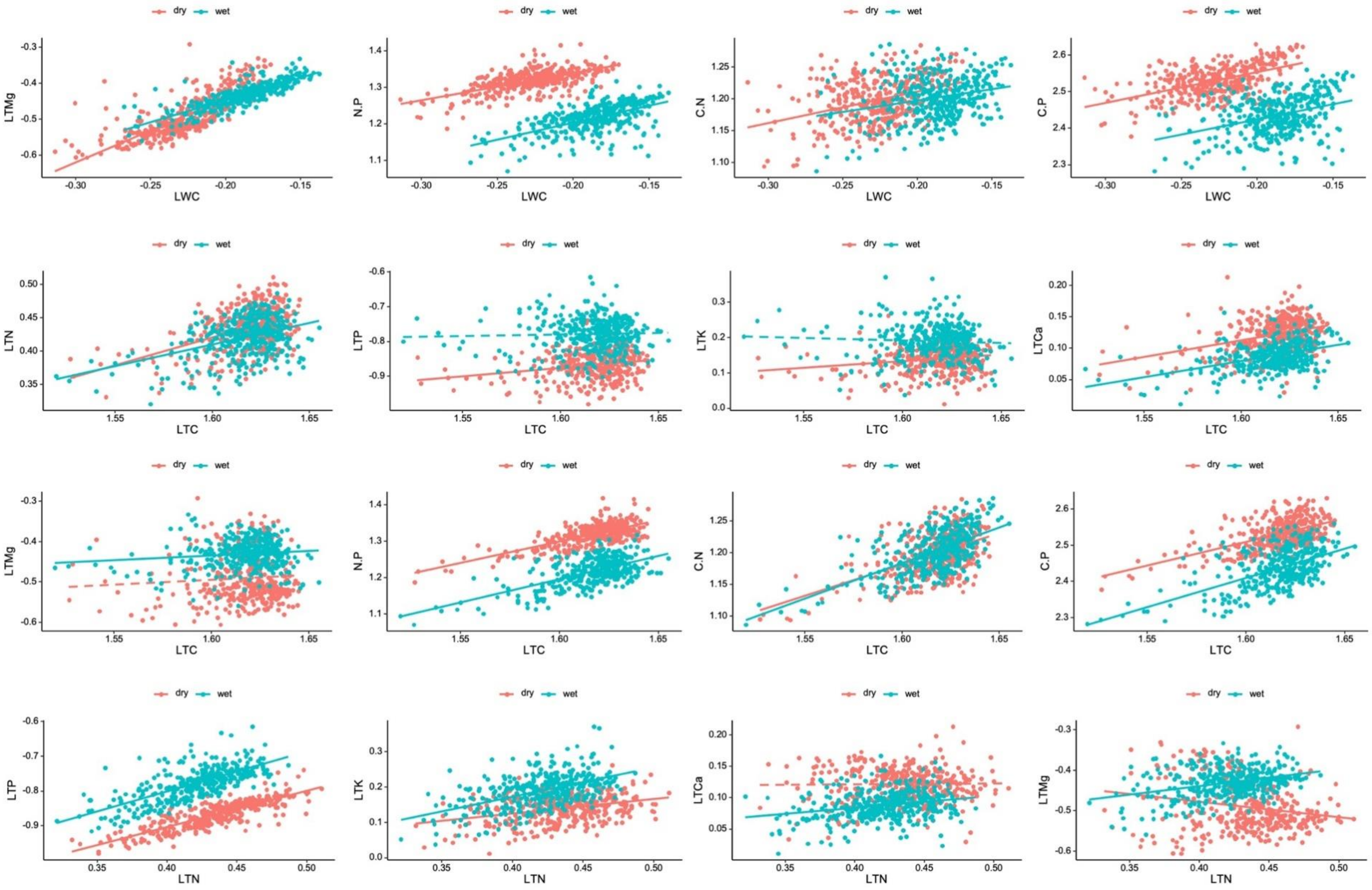
dry
wet
LTMg
N.P
C.N
C.P
LWC
LTN
LTP
LTK
LTCa
LTC

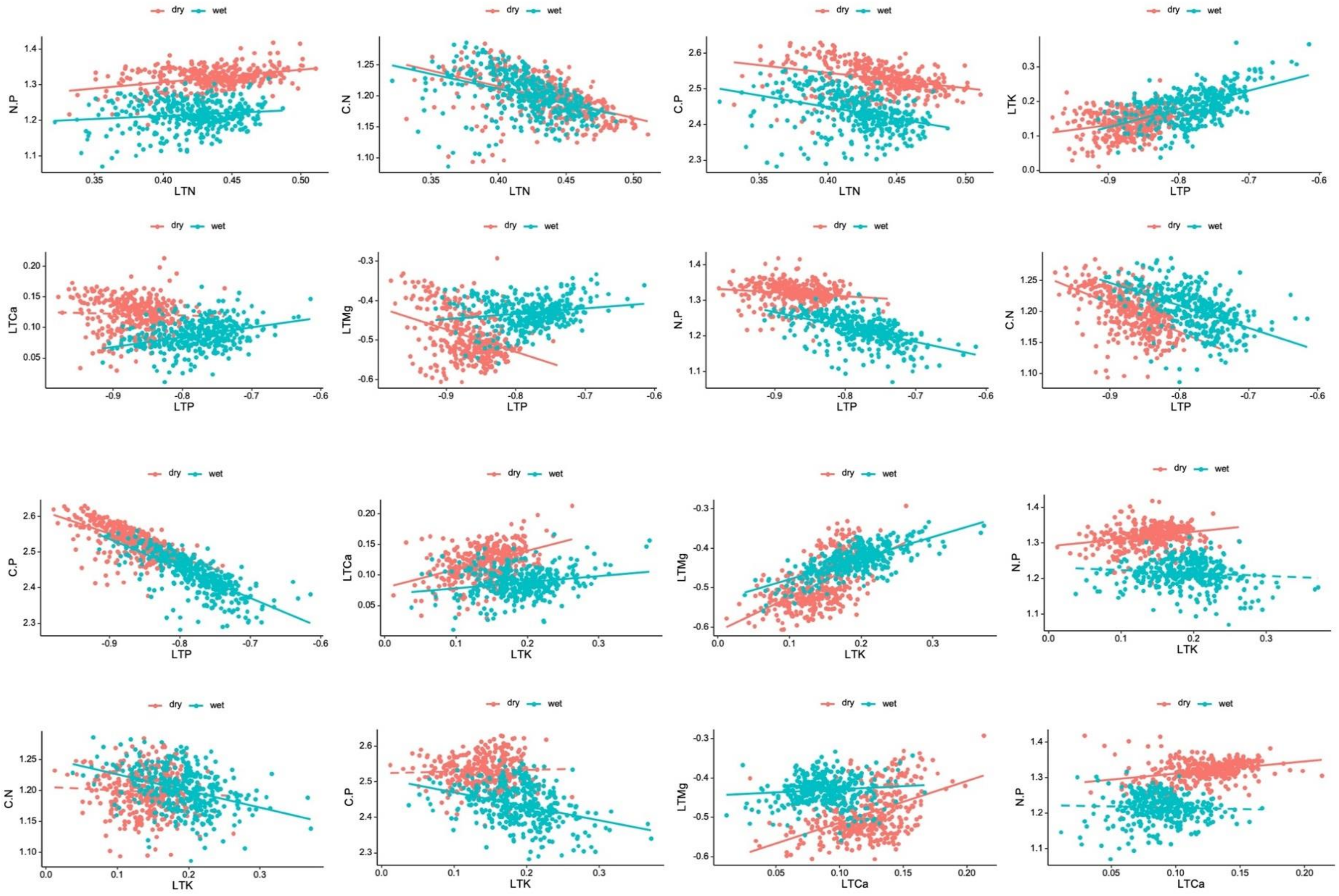
dry wet
N.P
LTN
C.N
LTN
C.P
LTN
LTK
LTP
LTCa
LTP
LTMg
LTP
N.P
LTP
C.N
LTP
C.P
LTP
LTCa
LTK
LTMg
LTK
N.P
LTK
C.N
LTK
C.P
LTK
LTMg
LTCa
N.P
LTCa

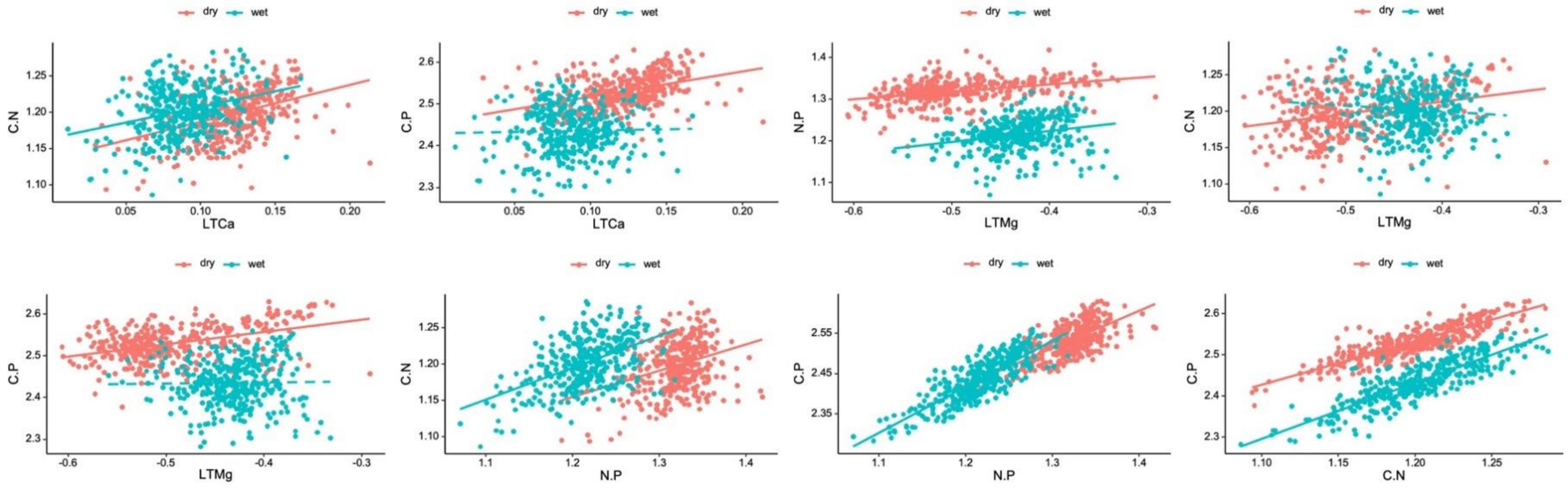

Fig. S2. Standardized major axis (SMA) relationships among CWM leaf functional traits of the 79 plant species in the 15-ha plot on Neilingding Island in the dry and wet seasons. Red dots and red lines indicate the dry season, and blue dots and blue lines indicate the wet season. Solid lines represent significant relationships and dashed lines represent nonsignificant relationships. Axes are log10-scaled.
